# Macrophage Extracellular Traps in Health and Disease: Current Concepts, Pathogenic Mechanisms and Clinical Implications

**DOI:** 10.3390/cells15141242

**Published:** 2026-07-09

**Authors:** Bojan Stojanovic, Ivana Milivojcevic Bevc, Bojana S. Stojanovic, Milica Dimitrijevic Stojanovic, Nenad Zornic, Ana Lukovic, Nikola Mirkovic, Strahinja Krsmanovic, Jelena Nesic, Danijela Tasic-Uros, Stefan Jakovljevic, Aleksandar Matic, Stevan Eric, Tomislav Nikolic, Jasmina Stojanovic, Nikola Prodanovic, Mladen Pavlovic

**Affiliations:** 1 Department of Surgery, Faculty of Medical Sciences, University of Kragujevac, 34000 Kragujevac, Serbia; bojan.stojanovic01@gmail.com (B.S.);; 2 Center for Molecular Medicine and Stem Cell Research, Faculty of Medical Sciences, University of Kragujevac, 34000 Kragujevac, Serbia; 3 Institute for Emergency Medicine Belgrade, 11000 Belgrade, Serbia; 4 Department of Pathophysiology, Faculty of Medical Sciences, University of Kragujevac, 34000 Kragujevac, Serbia; 5 Department of Pathology, Faculty of Medical Sciences, University of Kragujevac, 34000 Kragujevac, Serbia; 6 Department of Otorhinolaryngology, Faculty of Medical Sciences, University of Kragujevac, 34000 Kragujevac, Serbia; 7 Department of Internal Medicine, Faculty of Medical Sciences, University of Kragujevac, 34000 Kragujevac, Serbia

**Keywords:** macrophage extracellular traps, METs, METosis, extracellular traps, macrophages, inflammation, sterile inflammation, autoimmunity, fibrosis, cancer

## Abstract

**Highlights:**

**What are the main findings?**

- Macrophage extracellular traps are distinct, chromatin-based immune structures that extend macrophage function beyond phagocytosis, cytokine release, and antigen presentation, with context-dependent roles in antimicrobial defense and tissue signaling.
- MET formation is regulated by multiple, partly overlapping mechanisms, including PAD-dependent histone citrullination, reactive oxygen species, calcium signaling, mitochondrial DNA release, extracellular DNA sensing, protease activity, and macrophage–stromal cell crosstalk.

**What are the implications of the main findings?**

- METs may be protective when they support pathogen containment and early innate immune defense, but excessive or unresolved MET formation can amplify sterile inflammation, autoimmunity, fibrosis, metabolic dysfunction, vascular injury, organ damage, and cancer progression.
- Understanding MET biology as a macrophage-specific and tissue-dependent process may help identify new biomarkers and therapeutic targets, including strategies aimed at limiting harmful trap formation, promoting trap clearance, or modulating downstream inflammatory pathways.

**Abstract:**

Macrophage extracellular traps (METs) are chromatin-based structures released by activated macrophages and are increasingly recognized as distinct, context-dependent effectors of innate immunity. Although initially described in antimicrobial defense, METs are now implicated in sterile inflammation, autoimmunity, fibrosis, metabolic and vascular injury, organ-specific damage, and cancer. This review integrates dispersed evidence on MET biology across physiological and pathological settings, moving beyond neutrophil-centered interpretations of extracellular trap biology. We summarize the molecular composition, structural heterogeneity, major forms of METosis, and key regulatory pathways, including PAD-dependent chromatin remodeling, reactive oxygen species and calcium signaling, mitochondrial DNA release, extracellular DNA sensing, protease-mediated injury, and macrophage–stromal crosstalk. We also discuss the dual nature of METs as protective structures that can contain pathogens and amplify early innate responses, but also as pathogenic platforms when excessive, persistent, or insufficiently cleared. Overall, current evidence supports METs as functionally versatile macrophage-derived immune structures whose biological effects depend on the stimulus, tissue microenvironment, and disease context. By providing a unified framework, this review highlights the relevance of METs as potential biomarkers and therapeutic targets in inflammatory, fibrotic, vascular, autoimmune, and malignant diseases.

## 1. Introduction

Macrophage extracellular traps (METs) have emerged as an important yet still incompletely integrated component of innate immunity, extending the functional repertoire of macrophages beyond phagocytosis, cytokine secretion, and antigen presentation into the realm of extracellular chromatin-based defense and tissue signaling [1,2]. Since their first description in 2010, MET research has expanded from antimicrobial defense to a broad range of sterile and disease-associated inflammatory settings [2,3]. Despite the rapid expansion of the field, current knowledge remains fragmented across infection-specific studies, organ-centered disease models, and mechanistic reports, while the broader extracellular-trap literature is still largely shaped by neutrophil-centered paradigms that do not fully reflect macrophage-specific biology [4].

Historically, extracellular trap biology emerged from neutrophil research before METs were formally recognized as macrophage-derived structures [5]. In 1996, Takei and colleagues described PMA-induced rapid human neutrophil death with morphological features distinct from typical apoptosis or necrosis, including nuclear changes that were later interpreted within the conceptual framework of NETosis [5]. The antimicrobial identity of neutrophil extracellular traps was established in 2004, when Brinkmann and colleagues showed that activated neutrophils release chromatin fibers decorated with granular proteins that bind bacteria, degrade virulence factors, and support extracellular microbial killing [6]. Subsequent work defined NETosis as a regulated ROS-dependent cell death program distinct from apoptosis and necrosis, thereby providing the first mechanistic basis for understanding extracellular trap release as an active immune process rather than passive DNA leakage [7]. This historical trajectory is directly relevant to MET biology because macrophage trap formation shares several core cellular principles with NETosis, including danger sensing, chromatin decondensation, nuclear barrier disruption, DNA–protein scaffold assembly, and extracellular release. However, METosis should not be reduced to a macrophage copy of NETosis, because macrophage origin, tissue niche, PAD2/PAD4 usage, mitochondrial DNA contribution, viability status, and downstream inflammatory consequences may differ substantially from neutrophil-centered models.

This review integrates current evidence on MET biology and emphasizes the mechanisms that distinguish macrophage-derived traps from neutrophil-centered models of extracellular trap formation. Particular emphasis is placed on the features that define MET biology, including cellular origin, molecular composition, structural heterogeneity, modes of METosis, upstream triggers, and context-dependent functional consequences.

## 2. Macrophages in Host Defense, Tissue Maintenance, and Repair

Macrophages are highly versatile innate immune cells that operate at the interface between tissue homeostasis, host defense, and repair [8,9]. Distributed throughout virtually all tissues, they act as local sentinels that continuously survey the microenvironment, remove apoptotic cells, cellular debris, and harmful stimuli, and thereby preserve tissue integrity under steady-state conditions [10]. Following infection or sterile injury, macrophages are among the earliest responders: they recognize pathogen- and damage-associated signals through pattern-recognition receptors, initiate phagocytic clearance, and rapidly produce cytokines, chemokines, and other mediators that shape the subsequent inflammatory response [11,12]. Beyond their role in immediate defense, macrophages also link innate and adaptive immunity through antigen processing and presentation, while their remarkable functional plasticity allows them to either amplify inflammation or promote its resolution, depending on contextual cues from the tissue niche [13]. In parallel, they support regeneration by coordinating extracellular matrix remodeling, angiogenesis, and restoration of tissue architecture [14].

Beyond these classical functions, macrophages can release METs as an extracellular response to selected microbial and sterile stimuli [15]. This concept was first introduced in 2010, when Chow and colleagues demonstrated that mature macrophages, including RAW 264.7 cells and primary murine peritoneal macrophages, could be induced by *Staphylococcus aureus* and phorbol 12-myristate 13-acetate to generate extracellular traps, thereby extending trap biology beyond neutrophils [2,3]. Subsequent experimental work further supported the view that METs represent a genuine macrophage effector program, showing that macrophages can release trap-like structures in response to distinct microbial stimuli, including *Escherichia coli*, *Candida albicans*, and mycobacteria, although the antimicrobial efficiency of these structures appears to be stimulus- and pathogen-dependent [16]. Thus, MET formation represents an inducible macrophage effector response whose consequences depend on the stimulus and tissue environment.

### 2.1. Molecular Composition and Structural Forms of METs

Macrophage extracellular traps are extracellular, chromatin-based supramolecular networks released by activated macrophages in response to both infectious and sterile stimuli [17]. Their structural backbone is formed by decondensed extracellular DNA associated with histones, creating a scaffold that is further enriched with a broad range of bioactive proteins [18]. Similarly to neutrophil extracellular traps (NETs), METs contain double-stranded DNA (dsDNA), histones, myeloperoxidase (MPO), elastase, and lysozyme, but they may also incorporate additional macrophage-related components, including cluster of differentiation 68 (CD68), matrix metalloproteinase-9 (MMP-9), matrix metalloproteinase-12 (MMP-12), NAD(P)H quinone oxidoreductase 1 (NQO1), lactoferrin, and citrullinated histones [4,19]. Histone citrullination, mediated primarily by peptidylarginine deiminase 2 (PAD2) and peptidylarginine deiminase 4 (PAD4), facilitates chromatin relaxation by reducing the positive charge of histones, thereby promoting extracellular trap formation [20]. Although nuclear DNA appears to represent the principal source of the extracellular scaffold in most settings, accumulating evidence indicates that mitochondrial DNA may also contribute to MET composition under certain conditions [21].

Across infectious and sterile contexts, the conserved structural core of METs consists of extracellular DNA and histones, whereas the relative enrichment with MPO, elastase, lysozyme, MMPs, CD68-associated material, citrullinated histones, and mitochondrial DNA varies according to macrophage source, activating stimulus, and tissue microenvironment [4,16,19]. Thus, differences between bacterial, fungal, and parasitic METs should be interpreted mainly as context-specific variations of a shared DNA–protein scaffold rather than as completely separate trap entities [19].

In addition to their complex molecular composition, METs display marked morphological heterogeneity, and their appearance may vary according to the triggering stimulus, cellular context, and stage of trap evolution. Rather than existing as a single uniform structure, METs can appear as diffuse bubble-like formations, comet-shaped projections, elongated filamentous strands, or reticular net-like arrays extending through the extracellular space [22,23,24]. Experimental observations indicate that these forms may change over time, suggesting that METs are dynamic rather than static structures [24]. For example, diffuse rounded structures may gradually develop into more elongated comet-like configurations, whereas in other settings METs adopt linear or interconnected web-like patterns linking adjacent cells [24]. Such structural diversity is likely to be biologically relevant, as it may influence the efficiency of microbial entrapment, the local concentration of antimicrobial mediators, and the extent of tissue interaction. Therefore, the morphological plasticity of METs should be regarded as an integral feature of their contribution to innate immune defense.

The identification of macrophage extracellular traps relies on a combination of morphological, molecular, and quantitative approaches that together ensure reliable detection and characterization. Ultrastructural visualization using scanning electron microscopy enables the direct observation of extracellular web-like structures emanating from macrophages, providing important spatial confirmation of trap formation [25]. Complementary to this, immunohistochemical and immunofluorescence techniques are widely employed to detect canonical MET components, including extracellular DNA, histones, and associated antimicrobial proteins [26]. These components are typically visualized using fluorescence or confocal laser microscopy, allowing precise localization and co-localization analyses within the extracellular space [26]. For quantitative assessment, MET formation is often expressed as the proportion of macrophages releasing traps across multiple microscopic fields, supported by image analysis platforms such as ImageJ, NetQuant, or DANA, which facilitate standardized and reproducible measurements [4]. In parallel, biochemical quantification of extracellular DNA release provides an additional layer of validation, commonly using fluorescent dyes such as SYTOX or PicoGreen, with signal intensity measured by fluorescence plate readers [4,27].

### 2.2. Signals That Promote and Restrain MET Formation

MET formation is induced by diverse microbial, inflammatory, metabolic, particulate, and damage-associated stimuli [28]. In most settings, MET generation begins with the sensing of pathogen-associated or damage-associated signals through pattern recognition receptors, which initiate intracellular programs that culminate in chromatin extrusion [29,30,31]. A variety of inflammatory mediators and non-microbial stimuli have been shown to promote this response, including tumor necrosis factor-α (TNF-α), interferon-γ (IFN-γ), interleukin-8 (IL-8), extracellular DNA, oxidative stress, and increased intracellular or extracellular calcium [32,33]. phorbol 12-myristate 13-acetate (PMA), hypochlorous acid (HOCl), lipopolysaccharide (LPS), palmitate, neutrophil elastase (NE), particulate matter, phosphatidylserine-rich microparticles, perfluorooctane sulphonate, monosodium urate crystals, extracellular cold-inducible RNA-binding protein (CIRP), and heme, have all been implicated as MET inducers [15,32,34,35]. Importantly, MET release generally intensifies with increasing stimulus concentration and longer exposure time, although this response eventually reaches a plateau, suggesting that trap formation is dynamically regulated and not unlimited [22,35].

Pathogens constitute one of the best-characterized groups of MET-inducing stimuli [15]. Macrophages derived from different tissues and anatomical compartments can release METs in response to a wide range of microorganisms, including Gram-positive and Gram-negative bacteria, mycobacteria, fungi, yeasts, and parasites [2]. Reported inducers include *Mycobacterium tuberculosis*, *Candida albicans*, *Porphyromonas gingivalis*, *Mycoplasma species*, *Schistosoma japonicum*, and *Neospora caninum* [22,23,36,37,38]. However, the pathways through which pathogens promote MET formation are not uniform and may depend on the specific virulence factors and host signaling mechanisms involved [2]. For example, MET induction by *Mycobacterium tuberculosis* has been linked to 6-kDa early secretory antigenic target (ESAT-6), a key virulence-associated antigen of the *M. tuberculosis* complex, whereas *Porphyromonas gingivalis* appears to stimulate MET release through calcium influx associated with mitochondrial DNA extrusion [23,36]. These observations indicate that pathogen-driven METosis is mechanistically heterogeneous, with different microbes engaging distinct intracellular routes to achieve a similar extracellular defensive outcome. From this perspective, the most informative classification is not a strict division between aerobic and anaerobic pathogens, but the dominant intracellular route through which macrophages reach chromatin extrusion. Across pathogen classes, available data point to several recurrent modules: ROS/NADPH oxidase-dependent METosis, calcium- and PAD-associated chromatin remodeling, mitochondrial DNA-associated trap release, inflammasome/caspase-linked extracellular DNA extrusion, and autophagy-related MET formation [16,31,36]. For example, *Staphylococcus aureus*, *Clostridium perfringens*, *Bacillus* species, nontypeable *Haemophilus influenzae*, and *Mycoplasma bovis* illustrate ROS-linked MET responses, whereas Porphyromonas gingivalis, Pseudomonas aeruginosa under sulfated vizantin stimulation, and rapid-growing nontuberculous mycobacteria emphasize calcium-dependent or PAD-related programs. This indicates that pathogen-specific recognition events are diverse, but the downstream macrophage response converges on a limited number of chromatin-remodeling and trap-release programs [2,4].

In contrast, several factors have been shown to suppress MET formation, providing insight into the signaling pathways required for this process. Inhibition of superoxide generation significantly reduces MET release, underscoring the importance of oxidative signaling in at least a subset of MET programs [19,34]. Consistent with this, diphenylene iodonium and apocyanin, which interfere with reactive oxygen species production, have both been reported to inhibit MET formation [34]. Likewise, blockade of elastase activity with N-methoxysuccinyl-AAPC-CMK also attenuates trap release, suggesting that proteolytic remodeling contributes to chromatin extrusion or extracellular trap stabilization [34].

### 2.3. Molecular Pathways and Execution Steps of METosis

Macrophage extracellular trap formation, commonly referred to as METosis, is best understood as a regulated and context-dependent cellular program through which activated macrophages externalize chromatin structures decorated with antimicrobial and immunomodulatory proteins [2,15,28]. Rather than representing a single abrupt event, METosis proceeds as an ordered continuum that begins with the sensing of microbial or sterile danger signals through pattern-recognition and other upstream receptors, followed by activation of intracellular signaling networks involving redox changes, calcium flux, kinase cascades, and, in selected settings, inflammasome-related pathways [21,39,40]. These early signals commit the macrophage to a progressive remodeling program in which nuclear architecture loosens, chromatin becomes permissive to decondensation, and cytosolic or lysosome-associated effector proteins are mobilized toward the future extracellular scaffold [21,39]. In this way, METosis should be viewed not merely as extracellular DNA release, but as a coordinated transformation of the macrophage into a source of structured, biologically active chromatin-based defense material.

A pivotal early event in this process is chromatin decondensation, which is strongly influenced by the peptidylarginine deiminase axis. PAD2 and PAD4, both calcium-dependent enzymes expressed in macrophages, promote histone citrullination and thereby weaken histone–DNA interactions, allowing chromatin to relax and expand [18,20,39]. Current evidence suggests that PAD4 is particularly important for the generation of citrullinated histone-positive METs, whereas PAD2 also contributes substantially, although in a more context-dependent and sometimes partial manner [20,41]. This indicates that METosis is not driven by a single universal PAD program, but rather by a combinatorial mechanism whose exact composition depends on the stimulus, macrophage source, and tissue context [20,21]. In parallel, intracellular proteolytic processing may facilitate this step by increasing histone accessibility, although this remains less firmly established than the central role of histone citrullination itself [2,34]. Once chromatin relaxation has been initiated, decondensed nuclear DNA progressively mixes with proteins such as myeloperoxidase, elastase, and other inflammatory or antimicrobial components, while the nuclear envelope becomes destabilized and nuclear contents gain access to the cytoplasmic compartment [15]. This intermediate stage is crucial because it generates the DNA–protein scaffold that distinguishes bona fide METs from passive extracellular DNA released during nonspecific cell injury [21].

A pivotal early event in METosis is chromatin decondensation, a step that should be interpreted in light of both macrophage-specific observations and the more detailed mechanistic framework established in neutrophil ETosis [42,43]. In neutrophils, PAD4-mediated histone hypercitrullination reduces histone–DNA electrostatic interactions, promotes chromatin relaxation, and facilitates extracellular trap formation, providing a mechanistic model for the PAD-dependent chromatin remodeling observed in macrophages [42,43]. In macrophages, PAD4 appears particularly important for the generation of citrullinated histone-positive METs, whereas PAD2 may also contribute in a stimulus- and cell-source-dependent manner, indicating that the PAD axis is shared but not necessarily identical between NETosis and METosis [18]. After chromatin expansion, nuclear envelope rupture represents a second critical checkpoint because extracellular trap formation requires nuclear DNA to leave the nuclear compartment before it can assemble with cytoplasmic and antimicrobial proteins [44,45]. Recent NETosis studies indicate that this step is not merely passive nuclear disruption, but can involve kinase-regulated weakening of the nuclear lamina, including PKCα-mediated lamin B phosphorylation or disassembly and CDK4/6-associated lamin A/C phosphorylation during nuclear envelope breakdown [44,45]. Although direct evidence for identical lamin-kinase control in METosis remains limited, these findings provide a useful cellular framework for understanding how activated macrophages may transition from chromatin decondensation to nuclear content release without reducing the process to nonspecific necrotic DNA leakage. Once the nuclear barrier is breached, decondensed DNA can mix with myeloperoxidase, elastase, lysozyme, histones, matrix metalloproteinases, and other macrophage-associated inflammatory or antimicrobial proteins, generating the DNA–protein scaffold that distinguishes bona fide METs from passive extracellular DNA release [4]. The plasma membrane then represents the final physical barrier that determines whether the trap is released through lytic METosis or through more controlled, vital-like DNA export [18,21]. In lytic ETosis, studies in neutrophils show that plasma membrane breakdown is closely linked to cytoskeletal and endomembrane remodeling, with actin disassembly, ROCK–actomyosin signaling, and PKCα nuclear translocation contributing to the coordinated sequence that culminates in extracellular chromatin release [43,46]. For METosis, these observations support a staged execution model in which danger sensing and intracellular signaling are followed by PAD-dependent chromatin relaxation, lamin-associated nuclear envelope rupture, scaffold assembly, and final plasma membrane permeabilization or regulated DNA export. This distinction is important because it separates regulated METosis from accidental cell rupture and also explains why different stimuli may produce morphologically similar extracellular traps through partially different upstream routes [2]. Therefore, the lytic form of METosis should be viewed as a coordinated terminal program of chromatin externalization rather than as simple macrophage necrosis, while vital-like MET release remains a less completely defined process that requires further validation in macrophage-specific models [18].

At the mechanistic level, METosis can proceed through several partially overlapping upstream routes [15]. nicotinamide adenine dinucleotide phosphate oxidase (NADPH oxidase/NOX)-dependent and driven by reactive oxygen species (ROS) [2,47]. In this program, inflammatory or microbial stimuli activate macrophages, often via Toll-like receptors and related upstream signaling systems, leading to enhanced NADPH oxidase activity and ROS accumulation [2]. The resulting oxidative stress promotes chromatin relaxation, disruption of nuclear organization, and eventual extracellular trap release, frequently in association with membrane permeabilization [2]. This route has been implicated in several infections and inflammatory conditions, and its functional importance is supported by the finding that pharmacologic inhibition of NADPH oxidase attenuates MET formation [1,48]. However, METosis is not restricted to ROS-dependent execution. Alternative NADPH oxidase-independent pathways are increasingly recognized, particularly in response to Gram-negative bacteria, mycobacteria, and selected fungal or parasitic stimuli [36,49]. These non-canonical routes may rely more strongly on intracellular calcium mobilization, PAD-mediated chromatin remodeling, autophagy-related signaling such as the adenosine monophosphate-activated protein kinase/Unc-51-like autophagy activating kinase 1/mechanistic target of rapamycin (AMPK/ULK1/mTOR) axis, or mitogen-activated protein kinase (MAPK)-dependent programs, including extracellular signal-regulated kinase 1/2 (ERK1/2) and p38 MAPK signaling [4,39]. Additional modulators, including perturbation of the sterol biosynthetic pathway and cluster of differentiation 18 (CD18)-linked responses to microbial toxins, and mitochondrial stress, further reinforce the concept that METosis is mechanistically heterogeneous and governed by a network of intersecting molecular programs rather than by a single canonical pathway [2,4].

At the execution stage, METosis is generally described as proceeding through two principal forms, lytic and vital-like, although the boundary between them is not always absolute [18,21]. Lytic METosis is characterized by progressive membrane permeabilization and terminal cellular injury, culminating in the extracellular release of chromatin together with associated antimicrobial and inflammatory proteins [1,35]. In selected inflammatory settings, this pathway involves canonical or non-canonical inflammasome activation, followed by caspase-mediated cleavage of gasdermin D and generation of its pore-forming N-terminal fragment, which disrupts ionic homeostasis and facilitates expulsion of the preassembled MET scaffold [35,50]. If membrane injury progresses beyond a reversible threshold, plasma membrane rupture follows and bulk chromatin is discharged extracellularly [21]. By contrast, vital-like METosis refers to a non-lytic mode of extracellular trap release in which macrophages extrude DNA while temporarily preserving membrane integrity and short-term viability [21]. In this model, nuclear DNA is thought to exit through localized membrane discontinuities or pore-permissive routes and then undergo vesicular trafficking through the cytoplasm before extracellular export [21]. Reports of focal nuclear lamina discontinuities, transient membrane permeabilization, and endosomal sorting complexes required for transport (ESCRT)-dependent membrane-repair mechanisms suggest that some macrophages may enter an intermediate state in which DNA release and temporary survival coexist [21,51]. These observations should be placed within the broader stepwise model of ETosis, in which chromatin decondensation is followed by nuclear lamina weakening, nuclear envelope rupture, cytoskeleton and endomembrane remodeling, and either terminal plasma membrane breakdown or transient membrane damage followed by repair [42]. Live-cell imaging of NETosis has shown that cytoskeletal and endomembrane disassembly precede final chromatin externalization, while ROCK–actomyosin signaling can regulate PKCα nuclear translocation and thereby connect cytoskeletal remodeling with nuclear envelope rupture [43]. For METosis, this supports the interpretation that vital-like DNA release may represent an intermediate state between fully lytic trap formation and preserved macrophage viability, but the exact molecular machinery still requires macrophage-specific validation. Accordingly, the distinction between lytic and vital-like METosis is best viewed as an operational and context-dependent continuum rather than a rigid binary classification.

**Figure 1:**
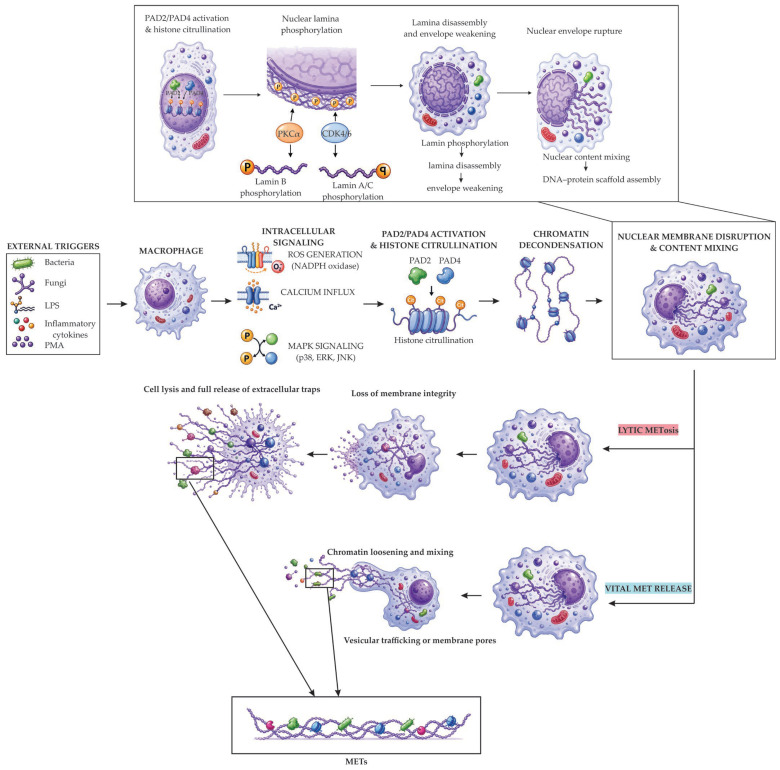
Molecular pathways and execution modes of macrophage extracellular trap formation. The figure summarizes the main stages of MET formation, including external stimulation, intracellular signaling, PAD-dependent histone citrullination, chromatin decondensation, nuclear membrane disruption, and lytic or vital-like MET release. The upper inset illustrates the proposed sequence of nuclear envelope rupture during METosis, showing PAD2/PAD4 activation and histone citrullination, nuclear lamina phosphorylation, lamina disassembly and envelope weakening, nuclear envelope rupture, and nuclear content mixing with DNA–protein scaffold assembly. PKCα-mediated lamin B phosphorylation and CDK4/6-associated lamin A/C phosphorylation are presented as kinase-regulated mechanisms that may contribute to nuclear lamina destabilization and nuclear envelope breakdown. Following nuclear envelope disruption, decondensed chromatin mixes with cytoplasmic and antimicrobial proteins, including MPO, elastase, lysozyme, histones, and MMPs, before extracellular release. The figure also distinguishes lytic METosis, characterized by loss of membrane integrity and complete extracellular trap release, from vital-like MET release, which may involve vesicular trafficking or membrane pores. Abbreviations: METs, macrophage extracellular traps; LPS, lipopolysaccharide; PMA, phorbol 12-myristate 13-acetate; ROS, reactive oxygen species; NOX/NADPH oxidase, nicotinamide adenine dinucleotide phosphate oxidase; MAPK, mitogen-activated protein kinase; ERK, extracellular signal-regulated kinase; JNK, c-Jun N-terminal kinase; PAD2/4, peptidylarginine deiminase 2/4; PKCα, protein kinase C alpha; CDK4/6, cyclin-dependent kinase 4/6; MPO, myeloperoxidase; MMPs, matrix metalloproteinases; DNA, deoxyribonucleic acid.

Finally, although nuclear chromatin is the principal scaffold of METs in most settings, mitochondrial DNA may also contribute under conditions of mitochondrial stress [28]. Regulated mitochondrial permeabilization through mechanisms involving BCL-2-associated X protein (BAX)/BCL-2 antagonist/killer 1 (BAK), voltage-dependent anion channel (VDAC)-associated channels, or mitochondrial permeability transition pore (mPTP)-related pathways can increase the escape of mitochondrial nucleic acids, particularly mitochondrial DNA (mtDNA), into the cytosol before their extracellular extrusion [28,52]. Because mitochondrial DNA is often oxidized and rich in immunostimulatory cytosine-phosphate-guanine (CpG) motifs, its incorporation into METs may influence both the structural composition and inflammatory potential of the resulting extracellular traps [28,52]. Together, these findings define METosis as a multistep program that links danger sensing, chromatin remodeling, and regulated DNA export. The multistep sequence of MET formation, from external triggering stimuli and intracellular signaling to chromatin remodeling and lytic or vital-like MET release, is summarized in Figure 1.

### 2.4. Macrophage Polarization, Phagocytosis, and Autophagy as Determinants of MET Formation

Macrophage polarization appears to be a major determinant of MET formation, indicating that extracellular trap release is closely linked to the functional state of these cells rather than representing a uniform macrophage response [2,19]. Current evidence suggests that METs are generated predominantly by classically activated, M1-polarized macrophages, which are characterized by a pro-inflammatory phenotype and by the production of cytokines such as interleukin-1 (IL-1), interleukin-6 (IL-6), interleukin-12 (IL-12), interleukin-23 (IL-23), and TNF-α [13,19]. This inflammatory milieu, together with canonical M1-polarizing stimuli including IFN-γ, lipopolysaccharide, and TNF-α, activates signaling pathways that favor METosis, particularly those involving reactive oxygen species generation and calcium influx [53]. Because M1 macrophages are more prone to oxidative burst and inflammatory signal integration, they are more likely to undergo extracellular trap formation and to release METs enriched in bactericidal and tissue-remodeling effectors such as myeloperoxidase (MPO) and matrix metalloproteinase-12 (MMP-12) [39,53]. By contrast, alternatively activated M2 macrophages exhibit a markedly lower tendency to release METs, consistent with their anti-inflammatory and tissue-repair-oriented profile [32]. Importantly, this relationship is not unidirectional, since METs themselves may further reinforce M1 polarization, thereby creating a positive feedback loop that sustains inflammatory amplification and may aggravate disease progression in chronic pathological settings [15].

METosis is also tightly integrated with other core macrophage functions, particularly phagocytosis and autophagy, which indicates that extracellular trap release should be viewed as part of a broader macrophage response program rather than an isolated event [15]. In some contexts, MET formation appears to cooperate with phagocytosis [4,54]. For example, sperm-induced MET release is partially dependent on phagocytic activity, since inhibition of phagocytosis reduces both sperm uptake and subsequent trap formation, suggesting that internalization of a target may help initiate or potentiate METosis [54]. In other situations, however, METosis may compensate for the limitations of phagocytosis, as illustrated by phosphatidylserine-rich microparticles, where larger particles induce greater trap release, possibly because they are less efficiently cleared by engulfment alone [55]. Autophagy represents another important regulator of this process. In macrophages exposed to aflatoxin B1, induction of autophagy parallels MET formation, whereas pharmacologic inhibition of autophagy suppresses trap release, supporting an autophagy-dependent mode of METosis in that setting [56].

In summary, METosis should be viewed as an inducible macrophage program shaped by activation state, stimulus type, and tissue context. The available evidence indicates that METosis is not a single uniform process, but a regulated and context-dependent continuum shaped by macrophage activation state, microbial or sterile danger signals, oxidative stress, calcium flux, PAD2/PAD4-mediated histone citrullination, autophagy-related pathways, MAPK signaling, inflammasome–gasdermin D activity, and, in selected settings, mitochondrial DNA release. Structurally, METs are extracellular DNA–protein networks enriched with histones, antimicrobial enzymes, inflammatory mediators, and macrophage-associated components, with morphology ranging from diffuse extracellular chromatin to filamentous and web-like formations. Functionally, METs may contribute to antimicrobial defense by immobilizing pathogens and concentrating effector molecules, but the same mechanisms can also amplify sterile inflammation, reinforce M1 polarization, aggravate tissue injury, and participate in chronic inflammatory, vascular, fibrotic, autoimmune, and malignant disorders. Therefore, METs should be viewed as a double-edged macrophage response: protective when spatially and temporally controlled, but potentially pathogenic when excessive, persistent, or insufficiently resolved, as summarized in Table 1.

## 3. Mets in Physiological Immune Protection

During early host defense, METs provide an extracellular mechanism for limiting pathogen spread when intracellular clearance is insufficient [2]. When macrophages encounter invading microorganisms or strong environmental danger cues, they may release extracellular chromatin scaffolds decorated with antimicrobial proteins that immobilize pathogens, restrict their local spread, and increase their exposure to concentrated effector molecules [18]. This function appears particularly relevant when microbes are too large, numerous, spatially clustered, or otherwise difficult to eliminate rapidly by phagocytosis alone, as may occur with bacterial aggregates, fungal elements, and selected parasites [22,47,49]. By creating a local meshwork around invading organisms, METs help contain infection within tissue compartments while buying time for the recruitment and coordination of additional innate immune cells [15,47]. In this way, the beneficial role of METs in health is not merely direct microbial killing, but also spatial containment of pathogens, reinforcement of early innate immune control, and support of an immediate protective inflammatory response that favors microbial clearance before dissemination occurs [47].

The pathogen-specific examples discussed below should therefore be read as variations of several shared principles rather than as unrelated responses to individual organisms [2,15]. First, METs are most consistently recruited when the infectious challenge is extracellularly accessible, clustered, persistent, too large, or insufficiently controlled by phagocytosis alone [15,22]. Second, the released traps usually perform one or more overlapping functions: physical immobilization of pathogens, restriction of local dissemination, concentration of antimicrobial proteins, and amplification of local inflammatory signaling [15]. Third, the same MET response may become harmful when it persists in vulnerable tissues, where extracellular DNA, histones, elastase, MPO, and MMPs can contribute to matrix injury, epithelial or membrane damage, and chronic inflammatory amplification.

### 3.1. METs in Host Defense Against Gram-Positive Bacteria

Gram-positive bacteria induce MET release through pathogen-specific mechanisms that differ in signaling requirements and functional consequences [1,15]. In response to organisms such as *Staphylococcus aureus*, *Streptococcus agalactiae*, and *Clostridium perfringens*, macrophages release DNA-based extracellular networks enriched with histones, myeloperoxidase, neutrophil elastase, and other antimicrobial mediators that immobilize bacteria, restrict their local dissemination, and enhance extracellular killing [25,57].

#### 3.1.1. Role of METs in Host Defense Against *Staphylococcus aureus*

*Staphylococcus aureus* is a major cause of persistent hospital-acquired infections, particularly in the setting of implanted medical devices, where biofilm formation promotes chronicity, recurrence, and resistance to clearance by conventional host defenses [58]. Upon exposure to *S. aureus*, macrophages release DNA-based traps enriched with antimicrobial effectors such as myeloperoxidase and elastase. Upon exposure to *S. aureus*, macrophages can release DNA-based extracellular traps enriched with antimicrobial effectors such as myeloperoxidase and elastase, which immobilize extracellular bacteria and enhance their killing in the local microenvironment [3,59]. Experimental studies further indicate that this response can be therapeutically amplified. Statins, through inhibition of 3-hydroxy-3-methylglutaryl-coenzyme A reductase (HMG-CoA reductase) and suppression of the sterol biosynthetic pathway, increase MET formation in RAW264.7 cells and murine peritoneal macrophages, thereby improving bacterial clearance both in vitro and in vivo; notably, this protective effect is abolished by excess mevalonate, supporting a mechanistic link with sterol pathway modulation [3]. A similar potentiating effect has been described for fosfomycin, which augments MET release in *S. aureus*-infected murine peritoneal macrophages and THP-1 cells through a NADPH oxidase-dependent ROS pathway, resulting in enhanced extracellular bactericidal activity [59]. The functional relevance of this pathway is underscored by the observation that inhibition of NADPH oxidase with diphenylene iodonium reduces bacterial killing, whereas blockade of elastase activity attenuates the antimicrobial efficacy of the traps [47].

#### 3.1.2. Role of METs in Host Defense Against *Streptococcus agalactiae*

*Streptococcus agalactiae*, or group B *Streptococcus* (GBS), is a major perinatal pathogen that colonizes the maternal genital tract and can ascend into gestational tissues, where it is associated with chorioamnionitis, preterm labor, preterm premature rupture of membranes, stillbirth, and neonatal sepsis [60]. At the maternal–fetal interface, placental macrophages mount a MET response when exposed to GBS, indicating that extracellular trap formation is part of the local innate defense program against this organism [25,61]. Mechanistically, GBS appears to trigger MET release through an oxidative burst–dependent pathway, most likely involving Toll-like receptor signaling, activation of NADPH oxidase, and reactive oxygen species generation, which then promote chromatin decondensation and extracellular DNA release [25]. These placental METs are decorated with histones, myeloperoxidase, neutrophil elastase, and, importantly, multiple matrix metalloproteinases, thereby combining direct antimicrobial activity with tissue-remodeling potential [25,61]. Functionally, this response is protective because the traps immobilize and kill GBS, and degradation of the extracellular DNA scaffold with deoxyribonuclease (DNase) reduces this bactericidal effect [25,61]. At the same time, the presence of matrix metalloproteinase (MMP)-rich METs gives this response a clinically important dual character: while MET formation may help contain ascending infection, excessive or sustained release could also contribute to extracellular matrix breakdown within fetal membranes and thereby link antimicrobial defense to membrane weakening and adverse pregnancy outcomes [25].

#### 3.1.3. Role of METs in Host Defense Against *Clostridium perfringens*

*Clostridium perfringens* is a Gram-positive, anaerobic, spore-forming pathogen that colonizes humans and animals and is best known as a cause of acute enterotoxemia and life-threatening gas gangrene, a rapidly progressive infection marked by extensive tissue destruction and high mortality [62]. In this setting, METs appear to represent an important protective component of innate immune defense. Upon exposure to *C. perfringens*, bone marrow-derived macrophages and J774.A1 macrophages release extracellular DNA structures decorated with histone H3, myeloperoxidase, and neutrophil elastase, thereby generating a bactericidal scaffold that contributes to microbial containment [57]. Mechanistically, this response depends on a coordinated signaling network involving NADPH oxidase-derived reactive oxygen species, ERK1/2 and p38 MAPK activation, store-operated calcium entry, PAD4 upregulation, and the participation of histones, MPO, and elastase [47,57]. These findings indicate that C. perfringens-induced MET formation is not simply the result of nonspecific macrophage injury, altered membrane permeability, or accidental extracellular DNA leakage, but rather reflects an actively regulated antimicrobial response involving intracellular signaling, chromatin remodeling, and DNA–protein scaffold assembly [47,57].

Importantly, the lack of dependence on lactate dehydrogenase release suggests that, in this model, MET extrusion is not primarily associated with overt lytic membrane rupture [57]. The biological relevance of this response is strongly supported by in vivo findings showing that DNase I-mediated degradation of extracellular traps impairs host protection during experimental gas gangrene, leading to increased bacterial burden, more severe tissue injury, and higher mortality [57].

#### 3.1.4. Role of METs in the Immunomodulatory Effects of *Bacillus subtilis* and *Bacillus licheniformis*

*Bacillus subtilis* and *Bacillus licheniformis* are members of the *B. subtilis* group that are widely recognized for their probiotic potential, largely because they produce enzymes and bioactive metabolites capable of suppressing the growth of pathogenic microorganisms and supporting host defense [63]. In this context, METs appear to represent one of the mechanisms through which these organisms enhance innate immune protection. Exposure of J774A.1 macrophages to either *B. subtilis* or *B. licheniformis* induces the release of METs through a ROS-dependent pathway, with the resulting extracellular traps characterized by the presence of myeloperoxidase and citrullinated histones, indicating active chromatin remodeling and antimicrobial programming [47,63]. These METs are not merely structural byproducts of macrophage activation, but functionally relevant effectors, as traps induced by *B. licheniformis* have been shown to exert sustained antimicrobial activity against several microorganisms, including *Staphylococcus aureus* [2,63]. The in vivo relevance of this response is further supported by murine studies demonstrating that these probiotic Bacillus species improve resistance to *S. aureus* infection in parallel with enhanced MET formation [2].

### 3.2. METs in Host Defense Against Gram-Negative Pathogens

Gram-negative infections remain a major cause of mucosal, pulmonary, gastrointestinal, genitourinary, and systemic inflammatory disease because these organisms combine invasive capacity with potent immunostimulatory surface components and, in many cases, marked resistance to host clearance [64]. In Gram-negative infection, MET formation is particularly useful for comparing ROS-dependent, calcium-dependent, and non-lytic forms of macrophage trap release [47].

#### 3.2.1. Role of METs in Host Defense Against *E. coli*

*Escherichia coli* is a common intestinal commensal, yet it also includes pathogenic strains capable of causing both intestinal and extraintestinal disease, particularly when host barriers are breached or virulence factors are expressed [65]. Experimental studies indicate that *E. coli* induces the formation of MET-like structures in murine macrophages through a process that is largely independent of NADPH oxidase-derived ROS and not associated with overt cell lysis, suggesting a non-lytic mode of extracellular trap release [16]. These structures contain nuclear DNA and mtDNA, together with antimicrobial molecules such as histones, MPO, and lysozyme. Their main role appears to be pathogen trapping rather than efficient bacterial killing. However, histones and cathelicidin-related antimicrobial peptide (CRAMP) may still contribute to some bactericidal activity [16,53]. From a mechanistic perspective, this pattern suggests alternative METosis pathways. These may include calcium signaling, PAD-mediated chromatin remodeling, and possibly autophagy-related AMP-activated protein kinase/Unc-51-like kinase 1/mechanistic target of rapamycin (AMPK/ULK1/mTOR) signaling [39]. Additional complexity is seen in uropathogenic *E. coli* (UPEC) infection, where hemolysin acts as a virulence factor that promotes MET formation in a dose- and time-dependent manner in both murine and human macrophages. The sensitivity of this response to DNase I, together with its resistance to cytochalasin D, supports a true extracellular DNA scaffold rather than a phagocytosis-dependent artifact [31]. In parallel, high burdens of *E. coli* may also trigger caspase-1-dependent extracellular trap release in human monocytes, indicating that inflammasome-associated programs can contribute in selected inflammatory contexts [66]. Clinically, these findings suggest that METs may have a protective role in *E. coli* infection by helping to contain extracellular bacteria and limit systemic spread. However, their effects are not always beneficial. In the urogenital tract, excessive trap formation may impair sperm motility and could link chronic genital inflammation, infection, and male infertility [54,67].

#### 3.2.2. Role of METs in Host Defense Against *Salmonella Typhimurium*

*Salmonella enterica* serovar Typhimurium is an important zoonotic intracellular pathogen and a major cause of enteric disease in both humans and animals, with the capacity to invade tissues, survive within host cells, and disseminate beyond the intestinal compartment [68]. In response to this challenge, macrophages deploy METs as an early extracellular defense mechanism that complements intracellular killing by rapidly trapping bacteria within DNA-based scaffolds and restricting their motility and local spread [69,70]. Experimental studies in J774A.1 macrophages and bone marrow-derived macrophages have shown that METs can be detected within minutes after infection, and their DNase sensitivity confirms that extracellular DNA is the structural core required for efficient bacterial entrapment [69]. Once formed, these structures reduce extracellular *Salmonella survival* through the local concentration of antimicrobial effectors associated with the traps, including histones and granule-derived enzymes such as myeloperoxidase and elastase [69]. At the mechanistic level, *Salmonella*-induced MET formation may be driven by bacterial surface components such as curli fibers. These highly inflammatory amyloid structures are involved in early bacterial aggregation and biofilm development. At the same time, *S. Typhimurium* has evolved countermeasures to weaken this response, including the extracellular nuclease 5′-nucleotidase, which degrades METs and thereby facilitates escape from macrophage-mediated extracellular killing [70].

#### 3.2.3. Role of METs in *Haemophilus influenzae* Infection

*Haemophilus influenzae*, particularly nontypeable *H. influenzae* (NTHi), is a major airway pathogen in chronic obstructive pulmonary disease, where persistent colonization and recurrent exacerbations drive hospitalization, accelerated lung-function decline, and mortality [71]. In this setting, macrophages respond to NTHi not only through phagocytosis but also by generating MET-like structures, indicating that extracellular trapping is recruited as an additional innate defense mechanism when bacterial persistence challenges conventional clearance [2,72]. From a mechanistic perspective, NTHi stimulates sustained reactive oxygen species production in bronchoalveolar macrophages, and this oxidant burst is closely linked to extracellular chromatin release, because inhibition of ROS with apocynin suppresses trap formation [72]. These NTHi-induced structures are not biologically inert. They are decorated with MMP-12, a macrophage metalloelastase linked to emphysema development and protease imbalance in chronic obstructive pulmonary disease (COPD). This suggests that the MET scaffold may help contain microbes, but may also carry tissue-damaging enzymes [2,72,73]. This dual role gives the response clear clinical relevance. On one hand, METs may help localize NTHi and support early airway defense by physically restricting bacterial dissemination within the infected microenvironment [2,72]. On the other hand, the same response may become harmful if it is repeatedly induced or insufficiently resolved, because NTHi-driven MET formation is associated with sustained ROS production and extracellular traps decorated with MMP-12 [72]. Since MMP-12 is a macrophage-derived metalloelastase implicated in protease–antiprotease imbalance, extracellular matrix degradation, and emphysematous remodeling, NTHi-induced METs may provide a local scaffold that concentrates tissue-injurious proteolytic activity in chronically inflamed airways [2,72,73]. This interpretation should be viewed as a mechanistic inference rather than definitive causal proof, because direct longitudinal evidence linking repeated NTHi-induced MET formation to progressive COPD tissue injury remains limited [2,72,73].

#### 3.2.4. Macrophage Extracellular Traps in *P. gingivalis*-Driven Inflammation

*Porphyromonas gingivalis* (*P. gingivalis*) is a key periodontal pathogen involved in chronic periodontitis. It expresses several virulence factors, including LPS, fimbriae, gingipains, and collagenase. Together, these factors promote persistent inflammation and progressive tissue destruction in the periodontal niche [74,75]. In response to this bacterium, macrophages can release METs in the form of extracellular web-like DNA structures decorated with myeloperoxidase, indicating activation of an extracellular antimicrobial program in addition to conventional phagocytic responses [36]. In terms of the underlying pathway, *P. gingivalis*-induced MET formation appears to be largely independent of NADPH oxidase-derived reactive oxygen species. This is supported by the finding that inhibition of ROS generation with diphenyleneiodonium (DPI) does not significantly reduce extracellular DNA release [36]. Instead, this response is strongly linked to intracellular calcium mobilization, since both intracellular and extracellular calcium chelation markedly suppress MET formation, identifying calcium influx as a central regulatory signal [36]. Notably, this pathway also appears to be independent of rapidly accelerated fibrosarcoma (RAF), mitogen-activated protein kinase kinase (MEK), and ERK signaling. This further supports the idea that *P. gingivalis* triggers a non-canonical form of MET formation [36]. From a functional perspective, these findings suggest that METs may support local antimicrobial defense by limiting bacterial persistence in periodontal tissues. However, during sustained infection, repeated calcium-driven trap release may also strengthen the inflammatory environment that contributes to periodontal tissue injury and disease progression [36].

#### 3.2.5. Role of METs in Host Defense Against *Pseudomonas aeruginosa*

*Pseudomonas aeruginosa* (*P. aeruginosa*) is a highly adaptable opportunistic Gram-negative pathogen. It causes severe airway, wound, and device-associated infections, and is especially important in cystic fibrosis. In this setting, biofilm formation and intrinsic antimicrobial resistance support persistence and make eradication difficult [76]. In this context, METs may act as an additional extracellular defense mechanism. They can support phagocytosis when macrophages encounter an organism that is difficult to clear [47,77]. Experimental work in RAW264.7 macrophages showed that sulfated vizantin, an immunomodulatory adjuvant, suppresses *P. aeruginosa* spread by promoting MET formation rather than by directly killing the bacterium [77,78]. At the cellular level, this response is linked to calcium influx, activation of PAD2, and histone citrullination. This indicates that chromatin remodeling is a key step in MET release in this model [77]. Importantly, blocking calcium entry with ethylenediaminetetraacetic acid (EDTA) or the T-type calcium channel blocker tetrandrine reduces MET formation. It also weakens the anti-dissemination effect of sulfated vizantin. These findings support a causal role for the calcium–PAD2 axis in this response [47,77].

### 3.3. MET-Mediated Responses to Mycobacteria and Other Bacteria

Beyond classical Gram-positive and Gram-negative pathogens, METs also participate in host responses to several other clinically important bacteria, particularly mycobacteria and atypical species that are difficult to eradicate by conventional macrophage mechanisms alone [2]. In these settings, METs appear to act mainly as an additional extracellular defense mechanism. They complement phagocytosis by trapping microorganisms, limiting their spread, and concentrating antimicrobial mediators within the infected microenvironment [1]. However, their biological significance is highly context-dependent, because in some infections METs contribute to microbial control, whereas in others they mainly reflect intense macrophage stress, tissue injury, or even pathogen-adapted immune evasion [79].

In *Mycobacterium tuberculosis* infection, MET formation occurs in human macrophages as part of the early response to a pathogen that has evolved highly effective intracellular survival strategies [30]. Alveolar and blood-derived macrophages exposed to *Mycobacterium tuberculosis* (*M. tuberculosis*) release extracellular DNA structures enriched with citrullinated histones. This response is strongly influenced by the early secretory antigenic target 6-kDa secretion system 1 (ESX-1) and its major virulence factor, 6-kDa early secretory antigenic target (ESAT-6) [23]. At the level of the underlying mechanism, ESX-1-dependent phagosomal injury and membrane damage appear to promote chromatin release, while elastase activity contributes to trap formation [30,80]. Interferon-γ further amplifies this process, enhancing both MET production and mycobacterial aggregation within macrophages, but at the same time it also increases ESX-1-associated necrosis [30,79]. Thus, in tuberculosis, METs should not be viewed as a purely protective response. Although they may help contain bacilli locally, current evidence suggests that they do not markedly reduce intracellular mycobacterial viability. Instead, they may reflect virulence-driven cell injury that supports persistence, immune dysregulation, and chronic infection [30,81].

Rapid-growing nontuberculous mycobacteria show a similarly complex relationship with METs. *Mycobacterium massiliense*, and related difficult-to-treat respiratory pathogens such as *M. abscessus* subsp. *massiliense*, induce MET release from macrophages through mechanisms that depend largely on calcium influx rather than NADPH oxidase activity [82]. The resulting structures contain both nuclear and mitochondrial DNA intertwined with histones, myeloperoxidase, and elastase, indicating that they are bona fide extracellular traps [47,82]. Their principal effect, however, seems to be physical containment rather than direct killing [79,82]. In some models, METs impede bacterial dissemination without clear bactericidal activity, whereas in others they may even support bacterial persistence or growth, underscoring that extracellular entrapment does not necessarily translate into microbial clearance [23,82].

By contrast, in *Mycoplasma bovis* infection, METs appear to play a more clearly beneficial antimicrobial role [48]. This pathogen, which is important in veterinary respiratory disease, induces bovine macrophages to release reticular extracellular structures composed of DNA fibrils decorated with histone H3, myeloperoxidase, neutrophil elastase, and several matrix metalloproteinases [48]. In this model, MET generation depends on NADPH oxidase activity and reactive oxygen species production, indicating engagement of a classical oxidative pathway of trap formation [48]. Importantly, MET release is associated with effective host-mediated bacterial killing, supporting the view that these structures are not simply byproducts of macrophage activation but active participants in immune defense.

**Figure 2:**
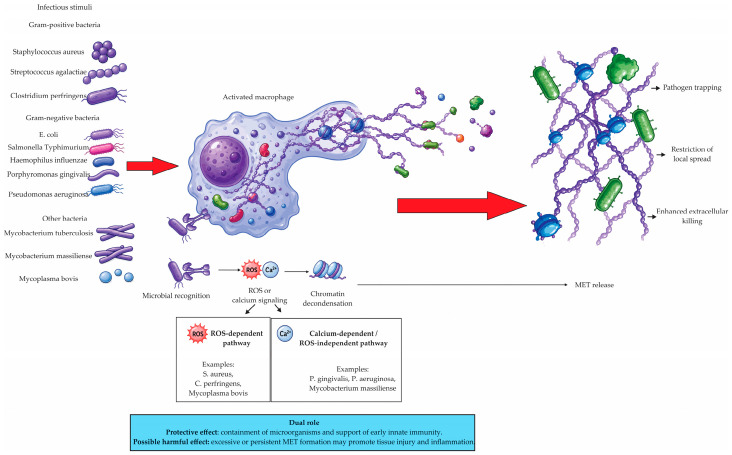
Macrophage extracellular traps in antibacterial host defense. The figure summarizes bacterial triggers of MET formation and the main downstream outcomes, including pathogen trapping, restriction of local spread, extracellular killing, and potential tissue injury during unresolved inflammation. Abbreviations: METs, macrophage extracellular traps; ROS, reactive oxygen species; Ca^2+^, calcium ion; PAD, peptidylarginine deiminase; DNA, deoxyribonucleic acid; *E. coli*, *Escherichia coli*; *S. aureus*, *Staphylococcus aureus*; *P. gingivalis*, *Porphyromonas gingivalis*; *P. aeruginosa*, *Pseudomonas aeruginosa*.

Figure 2 summarizes the main bacterial triggers, signaling routes, and functional outcomes of antibacterial MET formation. 

### 3.4. Role of METs in Host Defense Against Fungi

Fungal infections, especially those caused by *Candida albicans* (*C. albicans*), are a major challenge for innate immunity. Effective host defense requires not only fungal killing, but also containment of organisms that can invade tissues and evade immune responses [83]. In this context, macrophages contribute to antifungal defense by releasing METs after direct contact with fungal cells, and the magnitude of this response appears to increase with fungal burden [4,22]. These extracellular structures provide a DNA-based scaffold that captures *Candida* at the site of infection, thereby limiting tissue spread and reducing invasive potential, while in some settings also contributing to fungal killing through associated antimicrobial proteins [16,22]. However, the functional outcome of MET formation is not uniform across studies, as some data suggest that, in macrophages, their dominant role is containment rather than potent fungicidal activity [16]. This distinction is biologically relevant because *Candida albicans* can counteract trap-mediated defense by secreting DNases that degrade extracellular DNA, thereby weakening the structural basis of METs and facilitating escape [22]. Mechanistically, *Candida*-induced MET formation has been described as largely independent of the NADPH oxidase/ROS axis in some macrophage models, indicating that non-canonical signaling pathways can support antifungal trap release [16]. A related concept emerges from studies of fungal toxins, where aflatoxin B1 induces MET production in a dose-dependent manner through mechanisms linked to autophagy and ROS generation, and this response appears to contribute to toxin reduction [56].

### 3.5. Role of METs in Host Defense Against Parasites

Parasitic infections pose a distinctive challenge to innate immunity because many parasites are relatively large, motile, and poorly suited to rapid elimination by phagocytosis alone, making extracellular containment strategies particularly relevant [2]. In this context, METs appear to function as an additional macrophage defense mechanism that can immobilize parasites, concentrate antimicrobial mediators at the host–parasite interface, and, in some settings, directly promote parasite killing [2,84]. This has been shown most clearly for larval *Strongyloides stercoralis* (*S. stercoralis*). These larvae induce MET formation in human macrophages, and the resulting DNA-based structures contribute to larval entrapment and macrophage-mediated killing. However, this response appears to depend on the species and tissue context in murine models [49,84]. By contrast, some parasites can suppress this pathway. In schistosomiasis, extracellular vesicles released from *Schistosoma japonicum* (*S. japonicum*) eggs are enriched in *S. japonicum* microRNA-71a (Sja-miR-71a). This microRNA inhibits MET formation by targeting the semaphorin 4D/peroxisome proliferator-activated receptor gamma/interleukin-10 (Sema4D/PPAR-γ/IL-10) axis. As a result, macrophages shift toward an anti-inflammatory state, which may support immune evasion [85]. Protozoan parasites can also induce MET release through specific signaling pathways. For example, *Neospora caninum* (*N. caninum*) tachyzoites strongly promote MET formation in bovine macrophages through extracellular signal-regulated kinase 1/2 (ERK1/2)- and p38 mitogen-activated protein kinase (MAPK)-dependent cell death pathway [38].

In summary, METs represent a protective but finely balanced extension of macrophage antimicrobial activity, particularly in situations where phagocytosis alone is insufficient to control extracellular, clustered, large, or persistent pathogens. Across Gram-positive and Gram-negative bacterial infections, mycobacterial disease, fungal exposure, and parasitic invasion, METs mainly act by immobilizing microorganisms within extracellular chromatin scaffolds enriched with histones, myeloperoxidase, elastase, matrix metalloproteinases, and other antimicrobial or tissue-modifying proteins. Their biological value is most evident when trap formation limits microbial spread, enhances local killing, and supports early innate immune coordination; however, the same response may become clinically problematic when excessive or sustained, as illustrated by fetal membrane weakening in group *B. Streptococcus* infection, chronic airway matrix injury in nontypeable *Haemophilus influenzae* infection, periodontal tissue destruction in *Porphyromonas gingivalis*-driven inflammation, impaired reproductive function in urogenital infection, and pathogen persistence in tuberculosis and some nontuberculous mycobacterial infections. Thus, METs should not be interpreted simply as antimicrobial structures, but as context-dependent immune platforms whose protective or pathogenic outcome depends on the infecting organism, macrophage activation state, dominant signaling pathway, tissue niche, and efficiency of trap resolution; this integrated interpretation is summarized in Table 2.

## 4. METs in Disease

Under physiological conditions, METs contribute to host defense, but when their production becomes excessive, persistent, or insufficiently cleared, they may shift from a protective response to a driver of tissue injury and chronic disease [1,4]. In this setting, MET-derived extracellular DNA and histones can persist in the tissue microenvironment and act as potent endogenous danger signals, sustaining inflammatory cascades and amplifying immune activation [2,86]. One important consequence of this dysregulation is the generation of persistent DNA–autoantigen complexes, which may facilitate loss of immune tolerance and promote autoantibody formation, particularly when extracellular trap components are not efficiently removed [4,20]. At the tissue level, METs may worsen pathology through several overlapping mechanisms. These include activation of cyclic GMP-AMP synthase (cGAS)-dependent inflammatory signaling after uptake of cell-free DNA by nearby cells, direct cytotoxic effects of extracellular histones, and extracellular matrix (ECM) degradation by trap-associated proteases such as neutrophil elastase (NE) and MMPs [87,88]. In parallel, METs can increase cytokine and chemokine production. They may also sustain inflammatory macrophage or microglial polarization through pathways such as the cathelicidin LL-37/purinergic P2X7 receptor/nuclear factor κB (LL-37/P2X7R/NF-κB) axis. In this way, METs may promote persistent leukocyte recruitment and the shift from acute inflammation to chronic inflammatory injury [89].

In malignant disease, these same properties place METs within the broader network of inflammation-driven tumor promotion [15]. Chronic inflammation is a well-recognized enabling factor in carcinogenesis because it creates a microenvironment rich in cytokines, reactive species, growth-promoting signals, and anti-apoptotic cues, all of which favor genomic instability, continued cell survival, and tumor evolution [90]. Within the tumor microenvironment, macrophages, particularly tumor-associated macrophages, are highly plastic cells that integrate local signals and can adopt phenotypes that either restrain or support tumor growth [91]. Emerging evidence suggests that METs may contribute to this process. They may remodel the tumor immune microenvironment, promote ECM breakdown, and support the release of mediators involved in angiogenesis, invasion, and metastatic spread [15]. In this respect, METs may contribute to malignant progression not only by maintaining chronic inflammation but also by shaping the ecological conditions in which tumor cells interact with stromal and immune compartments [2]. At the same time, extracellular traps are not uniformly protumorigenic, since under certain conditions they may limit tumor cell proliferation or augment antitumor immune responses [15]. Thus, in cancer, the biological effect of METs appears to depend on the dynamic balance of tumor microenvironmental signals, macrophage heterogeneity, and the dominant signaling programs operating within a given malignancy.

### 4.1. Macrophage Extracellular Traps in Kidney Diseases

Kidney diseases are often characterized by sterile inflammation, oxidative stress, macrophage activation, and tissue injury, and growing evidence suggests that METs contribute to these processes [2]. In acute kidney injury, including ischemia/reperfusion injury and rhabdomyolysis-associated renal damage, MET formation appears to amplify inflammation and worsen tubular injury [92]. A similar pathogenic role has been proposed in crystal-related disease, where calcium oxalate or struvite crystals activate macrophages and promote inflammatory responses associated with MET release [93,94].

#### 4.1.1. Role of METs in Kidney Ischemia/Reperfusion Damage

Kidney ischemia/reperfusion injury (KIRI) is a major cause of acute kidney injury and is driven not by infection, but by a vigorous sterile inflammatory response that follows transient hypoxia and subsequent reperfusion, leading to tubular epithelial injury, endothelial dysfunction, leukocyte recruitment, and progressive loss of renal function [95]. Macrophages are central participants in this process, and recent evidence indicates that METs are not a secondary epiphenomenon but an active pathogenic component of renal injury [96,97]. In this setting, danger signals generated by ischemic tissue promote macrophage activation and METosis, with chromatin decondensation and extracellular trap release amplifying local inflammation through extracellular DNA, histone-associated cytotoxicity, and propagation of pro-inflammatory signaling within the injured kidney [96]. At the molecular level, this process appears to depend on the PAD4–citrullinated histone H3 (CitH3) axis. This links renal sterile inflammation with the core molecular machinery of trap formation [96]. This is clinically important because pharmacologic inhibition of METosis has now shown renoprotective potential: in a murine KIRI model, zafirlukast attenuated renal dysfunction and tissue injury while suppressing MET formation through downregulation of PAD4 and CitH3, supporting the concept that METosis is a meaningful therapeutic target rather than merely a biomarker of damage in ischemic acute kidney injury (AKI) [96].

#### 4.1.2. METs in Rhabdomyolysis-Induced Acute Kidney Injury

Rhabdomyolysis is a life-threatening syndrome caused by traumatic or non-traumatic skeletal muscle injury, in which myocyte breakdown leads to systemic release of myoglobin, heme, and other intracellular constituents that can precipitate acute kidney injury, one of its most serious and mortality-associated complications [98]. In both experimental and clinical settings, increasing evidence indicates that METs are actively involved in the pathogenesis of rhabdomyolysis-induced AKI [92,99]. Beyond the direct nephrotoxic effects of myoglobin and heme, renal injury is amplified by oxidative stress and inflammatory cell infiltration, particularly by macrophages accumulating within the kidney [100]. Mechanistically, heme-activated platelets generated during muscle necrosis interact with macrophage antigen-1 (MAC1), promoting intracellular reactive oxygen species production and histone citrullination, which together drive MET formation [99]. These extracellular traps, in turn, intensify acute renal inflammation and tissue injury, indicating that METosis functions as a pathogenic effector rather than a passive marker of macrophage activation [99]. The clinical relevance of this pathway is supported by the detection of METs and elevated circulating free DNA in patients with traumatic rhabdomyolysis [99]. Importantly, pharmacologic inhibition of trap formation with lactoferrin significantly reduced MET generation and ameliorated renal injury in glycerol-induced rhabdomyolysis models, highlighting the MAC1–ROS–histone citrullination–MET axis as a plausible therapeutic target for the prevention or attenuation of rhabdomyolysis-associated acute kidney injury [92,99].

#### 4.1.3. Role of METs in Kidney Stone Disease

Kidney stone disease is a common urological disorder in which both metabolic and infectious factors contribute to crystal deposition, local inflammation, and progressive renal injury [101]. Calcium-containing stones, particularly calcium oxalate calculi, account for the majority of cases and are increasingly recognized as active triggers of renal interstitial inflammation rather than inert mineral deposits [102]. In this setting, calcium oxalate crystals promote macrophage recruitment and activation, while crystal-stimulated tubular epithelial cells release exosomes that further amplify macrophage polarization toward a proinflammatory M1 phenotype and enhance MET formation [93]. At the signaling level, this process has been linked to the cyclic adenosine monophosphate (cAMP) response element-binding protein 1/CREB-regulated transcription coactivator 2 (CREB1/CRTC2)–exosomal microRNA-93-3p (miR-93-3p)–NF-κB-inducing kinase/nuclear factor κB2 (NIK/NF-κB2) axis. Through this pathway, epithelial–macrophage crosstalk may intensify crystal-induced tissue damage [93]. METs, in turn, likely aggravate renal injury by sustaining local inflammation and reinforcing the destructive microenvironment around crystal deposits [93]. A similar concept applies to infectious stones, particularly magnesium ammonium phosphate, or struvite, calculi, which arise in association with urease-producing bacteria such as *Proteus mirabilis* and *Klebsiella pneumoniae* [94]. Although traditionally viewed as the consequence of urine alkalinization and salt precipitation, these stones are now understood to reflect a more complex interaction between microbial infection, macrophage activation, and innate immune injury [103]. In this context, magnesium ammonium phosphate (MAP) crystals can induce macrophage pyroptosis, migrasome formation, and MET release. This suggests that METs are part of the inflammatory machinery linking crystal deposition to ongoing renal damage [94].

**Figure 3:**
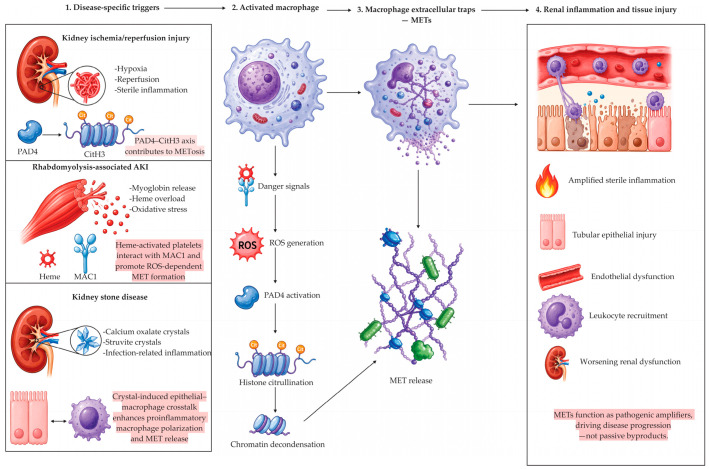
Macrophage extracellular traps in kidney disease. The figure summarizes how ischemic, toxic, heme-related, and crystal-associated renal stimuli may activate macrophages, promote MET formation, and amplify renal inflammation and tissue injury. Abbreviations: METs, macrophage extracellular traps; IRI, ischemia/reperfusion injury; AKI, acute kidney injury; PAD4, peptidylarginine deiminase 4; CitH3, citrullinated histone H3; ROS, reactive oxygen species; MAC1, macrophage-1 antigen; MAP, magnesium ammonium phosphate; CaOx, calcium oxalate.

The mechanisms by which kidney-specific injurious stimuli promote macrophage activation, MET formation, and subsequent renal inflammation and tissue injury are summarized in Figure 3.

### 4.2. METs in Respiratory Diseases

In respiratory diseases, METs are increasingly viewed as contributors to chronic airway and parenchymal inflammation rather than merely secondary byproducts of immune activation [104]. Across conditions such as severe asthma, COPD, cystic fibrosis, and pulmonary fibrosis, excessive MET formation appears to amplify inflammatory signaling, protease activity, and tissue remodeling, thereby promoting progressive lung injury and functional decline [2]. Overall, METs seem to link macrophage activation with persistent inflammation and structural damage in the lung.

#### 4.2.1. Macrophage Extracellular Traps in Asthma Pathogenesis

Asthma is a chronic inflammatory airway disease characterized by variable airflow limitation, immune dysregulation, and progressive structural remodeling in a subset of patients with severe disease [105]. Within this context, METs are increasingly recognized as amplifiers of airway inflammation, particularly in severe asthma, where classical monocytes and M1-polarized macrophages exhibit enhanced extracellular trap release [104,106]. Functionally, these M1-like METs promote inflammatory crosstalk by activating airway epithelial cells, neutrophils, and innate lymphoid cells (ILCs), especially ILC1 and ILC3. This facilitates inflammatory cell recruitment and increases cytokine production within the airway microenvironment [104,106]. Their abundance is positively associated with inflammatory markers, including monocyte chemoattractant protein-1 (MCP-1) and soluble ST2 (sST2), as well as with peripheral neutrophilia. In contrast, it is inversely related to lung function, as shown by lower predicted forced expiratory volume in 1 second (FEV1%) values in patients with severe asthma [104,106]. These findings suggest that METs are not passive biomarkers, but active contributors to persistent airway inflammation and possibly remodeling [104]. From a therapeutic perspective, inhibition of the IL-33/ST2 axis or blockade of PAD-dependent histone citrullination has been shown to reduce MET release, highlighting both pathways as promising targets [106]. Although DNase I can disrupt the extracellular DNA scaffold, it does not neutralize the histones and proinflammatory proteins embedded within METs, which may limit its overall efficacy [106].

#### 4.2.2. METs in Chronic Obstructive Pulmonary Disease

Chronic obstructive pulmonary disease (COPD) is a progressive inflammatory lung disorder characterized by persistent airflow limitation, small-airway remodeling, and emphysematous destruction that is most commonly driven by long-term exposure to cigarette smoke [107]. In this setting, METs appear to function not as a protective antimicrobial mechanism but as amplifiers of smoke-induced tissue injury [2]. Experimental studies have shown that cigarette smoke induces macrophage extracellular trap formation both in vitro and in vivo, in parallel with increased reactive oxygen species generation and enhanced expression of pathogenic proteases, including neutrophil elastase, MMP-9, and MMP-12 [108]. This is mechanistically important because COPD progression is closely linked to protease–antiprotease imbalance and extracellular matrix degradation, and METs provide a scaffold that concentrates these injurious mediators within the lung microenvironment [73,108]. As a result, MET formation may connect cigarette smoke exposure to persistent inflammation, proteolytic alveolar damage, and the development of emphysema [108]. The translational relevance of this pathway is supported by findings that DNase 1 reduces cigarette smoke-induced extracellular trap burden, macrophage accumulation, and lung proteolysis, suggesting that targeting extracellular DNA-based trap formation may represent a promising adjunct strategy for limiting inflammatory and tissue-destructive processes in COPD [108].

#### 4.2.3. METs in Cystic Fibrosis Lung Disease

Cystic fibrosis lung disease is characterized by recurrent lower respiratory tract infections, persistent neutrophil-dominated inflammation beginning early in life, and progressive structural airway damage culminating in bronchiectasis [109]. In this setting, METs appear to contribute to the chronic inflammatory burden and tissue injury that drive disease progression [34]. Bronchoalveolar samples from children with cystic fibrosis have demonstrated abundant formation of both NETs and METs, supporting the view that extracellular traps accumulate within the airway milieu and participate in local lung damage [34,110]. A key mechanistic link involves neutrophil elastase, a major determinant of bronchiectatic progression, which can be internalized by macrophages through endocytosis while retaining proteolytic activity [34]. Once internalized, neutrophil elastase promotes histone H3 citrullination and cleavage, thereby facilitating MET formation and amplifying airway inflammation [34]. This creates a self-reinforcing cycle in which neutrophilic inflammation promotes macrophage trap release, and METs in turn sustain protease-rich inflammatory injury [34]. These METs likely amplify airway inflammation by adding extracellular DNA, histones, and protease-associated signals to an already injurious microenvironment, thereby reinforcing mucus obstruction, inflammatory cell recruitment, and tissue damage [110]. From a clinical perspective, this makes the neutrophil elastase–macrophage extracellular trap axis particularly important. It links chronic infection and neutrophilic inflammation with macrophage-driven lung injury. Accordingly, DNase 1 and α1-antitrypsin (AAT) have been proposed as complementary strategies to reduce extracellular trap burden and protease-mediated damage in cystic fibrosis airways [34,110].

**Figure 4:**
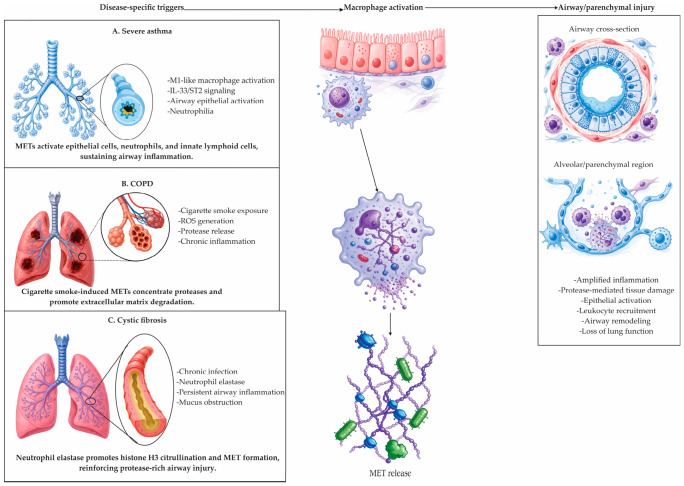
Macrophage extracellular traps in respiratory diseases. The figure summarizes the proposed contribution of METs to airway and parenchymal inflammation in asthma, COPD, cystic fibrosis, and pulmonary fibrosis. Abbreviations: METs, macrophage extracellular traps; COPD, chronic obstructive pulmonary disease; CF, cystic fibrosis; NE, neutrophil elastase; MMP, matrix metalloproteinase; MMP-12, matrix metalloproteinase-12; IL, interleukin; ST2, suppression of tumorigenicity 2; PAD, peptidylarginine deiminase.

The disease-specific triggers that promote macrophage activation and MET formation in severe asthma, COPD, and cystic fibrosis, together with their downstream contribution to airway and parenchymal injury, are summarized in Figure 4.

### 4.3. METs in Metabolic Diseases

Metabolic diseases are increasingly recognized as chronic inflammatory conditions in which macrophages actively shape tissue dysfunction, and METs appear to contribute to this process by amplifying sterile inflammation [2,111]. Evidence from obesity, type 2 diabetes, and atherosclerosis suggests that MET formation is linked to macrophage activation in metabolically stressed tissues, where it may worsen insulin resistance, sustain adipose inflammation, and promote vascular injury and thrombus organization [2]. Although the underlying mechanisms differ across disease settings, METs are emerging as a common immunopathological link between metabolic stress, chronic inflammation, and end-organ damage [2].

#### 4.3.1. MET-Mediated Mechanisms in Obesity

Obesity is increasingly recognized as a state of chronic low-grade inflammation in which visceral adipose tissue becomes infiltrated by macrophages that accumulate around dying adipocytes and form the characteristic crown-like structures [112]. These inflammatory foci are associated with activation of NF-κB and increased production of cytokines such as TNF-α and IL-1β, thereby creating a microenvironment that promotes sustained immune activation and metabolic dysfunction [113]. In this setting, METs appear to contribute to the amplification of adipose tissue inflammation. Experimental evidence indicates that TNF-α can directly stimulate macrophages to undergo METosis, while studies in obese mice suggest that this response occurs within crown-like structures through PAD-mediated histone citrullination, particularly involving PAD2-dependent modification of histone H4 [17]. Through this mechanism, METs may reinforce local inflammatory signaling and further aggravate adipose tissue injury [17]. The clinical significance of this process extends beyond local metabolic disturbance, because the proinflammatory mediators generated in obese adipose tissue are detectable systemically and have also been linked to obesity-associated complications, including breast cancer progression [114].

#### 4.3.2. METs in Type 2 Diabetes Mellitus

Type 2 diabetes mellitus is a chronic metabolic disorder characterized not only by hyperglycemia but also by persistent low-grade inflammation in adipose tissue and other insulin-sensitive organs, where macrophages play a central pathogenic role [115]. In this setting, METs are increasingly recognized as one mechanism by which activated macrophages amplify local inflammation and aggravate insulin resistance [4,116]. Experimental studies suggest that increased macrophage infiltration into adipose tissue is accompanied by enhanced MET formation, which further sustains the inflammatory microenvironment and interferes with insulin signaling [116]. This is biologically relevant because reduced MET formation in diabetic models has been linked to lower adipose tissue inflammation and improved insulin signaling. This includes restoration of insulin receptor substrate-1 (IRS-1), protein kinase B (AKT), and glycogen synthase kinase-3β (GSK-3β) pathways [2,116].

#### 4.3.3. Macrophage Extracellular Traps in Atherosclerotic Progression

Atherosclerosis is a chronic inflammatory disease of the arterial wall in which lipid deposition, macrophage infiltration, foam cell formation, and maladaptive vascular repair together drive plaque growth, luminal narrowing, and, ultimately, thrombotic complications [117]. Within this setting, METs are increasingly recognized as a relevant component of the macrophage contribution to plaque progression rather than merely a histologic by-product of inflammation [118]. Histopathologic studies of coronary lesions from patients with acute myocardial infarction have shown that, whereas NETs are more prominent in fresh thrombi, METs become particularly enriched in later, organized thrombi, suggesting that macrophage-derived traps may be especially important in thrombus maturation and stabilization [118]. Experimental work further supports an active role for METs in the atherosclerotic plaque microenvironment. Lipid-stressed foam macrophages can undergo chromatin decondensation and release extracellular traps. Emerging evidence links this process to PAD4-dependent histone citrullination, as well as upstream phosphoinositide 3-kinase/protein kinase B (PI3K/AKT) signaling and lipid mediators such as lysophosphatidylcholine (LPC) [2,119]. Functionally, these structures are well positioned to intensify vascular inflammation by providing extracellular DNA–protein scaffolds, concentrating proteolytic and pro-inflammatory mediators, promoting endothelial dysfunction, and reinforcing plaque instability and atherothrombotic progression [118,119]. Thus, current evidence supports the view that in atherosclerosis, METs may contribute less to the earliest initiation of plaque formation than to the maintenance of inflammatory plaque activity and the later organization of coronary thrombi, making METosis a potentially relevant target in advanced atheroinflammatory disease.

**Figure 5:**
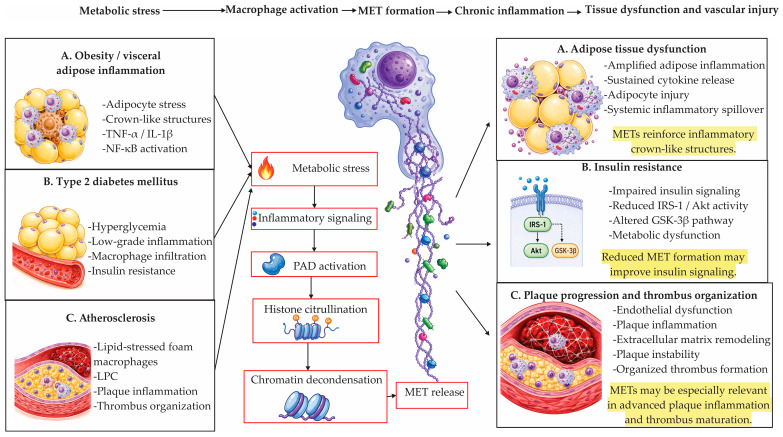
Macrophage extracellular traps as mediators of metabolic inflammation and vascular injury. The figure summarizes how metabolic stress, macrophage activation, and MET formation may contribute to adipose tissue inflammation, insulin resistance, endothelial dysfunction, plaque instability, and thrombus organization. Abbreviations: METs, macrophage extracellular traps; T2DM, type 2 diabetes mellitus; TNF-α, tumor necrosis factor-α; IL-1β, interleukin-1β; NF-κB, nuclear factor kappa B; IRS-1, insulin receptor substrate-1; AKT, protein kinase B; GSK-3β, glycogen synthase kinase-3 beta; LPC, lysophosphatidylcholine; PAD, peptidylarginine deiminase.

The proposed sequence linking metabolic stress, macrophage activation, MET formation, chronic inflammation, adipose tissue dysfunction, insulin resistance, and vascular injury is summarized in Figure 5.

### 4.4. METs in Autoimmune Diseases

Autoimmune diseases arise when mechanisms of self-tolerance fail and persistent innate immune activation is converted into chronic, self-directed inflammation, and METs are increasingly being recognized as one way this transition may be sustained at the tissue level [28]. Because macrophages normally regulate apoptotic cell clearance, antigen presentation, and local immune homeostasis, their shift toward extracellular trap release can transform them into a durable source of immunogenic DNA–protein complexes within inflamed organs such as synovium, kidney, lung, and mucosal tissues [2]. MET cargo is especially relevant in this context because it may contain citrullinated histones, oxidized mitochondrial DNA, and other modified intracellular molecules with strong autoantigenic potential. When these structures are insufficiently cleared, they prolong extracellular exposure of self-nucleic acids and post-translationally modified proteins, favor formation of stable autoantigen–DNA complexes, and promote autoantibody generation and fibro-inflammatory remodeling [20,28]. Current evidence therefore supports the view that in autoimmune disease, METs are not merely markers of inflammation but may function as tissue-embedded amplifiers that couple local stress to persistent nucleic-acid sensing, cytokine production, and loss of immune tolerance, although their precise contribution is likely disease- and organ-specific [28].

#### 4.4.1. Role of METs in the Immunopathogenesis of Rheumatoid Arthritis

Rheumatoid arthritis is a chronic autoimmune inflammatory disease characterized by persistent synovitis, progressive cartilage and bone destruction, and the emergence of autoreactive immune responses directed against citrullinated proteins [120]. In this setting, METs appear to contribute to disease pathogenesis by expanding the local pool of immunogenic citrullinated antigens and amplifying synovial inflammation [20,121]. Studies in collagen-induced arthritis and synovial tissue from patients with rheumatoid arthritis have shown that macrophages express functional PAD4, and likely PAD2 as well, enabling histone and extracellular protein citrullination during MET formation [20,121]. Through this process, macrophages release citrullinated chromatin and other modified antigens. These antigens may promote the formation of anti-citrullinated protein antibodies (ACPAs). In line with this, macrophage depletion reduces both the citrullination burden and serum ACPA levels, supporting a causal role of macrophage-driven trap formation in autoantibody development [20,121]. Beyond serving as a source of autoantigens, MET-derived DNA and associated cargo can directly activate fibroblast-like synoviocytes through cGAS-dependent signaling linked to PI3K/AKT activation, thereby promoting proliferative, invasive, and matrix-degrading phenotypes that resemble the aggressive stromal behavior typical of rheumatoid synovium [87,122]. These observations indicate that METs participate in rheumatoid arthritis on at least two pathogenic levels, by fueling the autoimmune response and by reprogramming the synovial microenvironment toward chronic tissue-destructive inflammation [20,87,121,123]. From a translational perspective, the reduction of citrullination and arthritis severity by PAD inhibition, together with emerging evidence supporting cGAS-STING blockade, suggests that targeting MET formation or downstream DNA-sensing pathways may represent a promising therapeutic strategy in rheumatoid arthritis.

#### 4.4.2. MET-Mediated Mechanisms in Systemic Lupus Erythematosus

Systemic lupus erythematosus (SLE) is a prototypical systemic autoimmune disease. It is driven by defective clearance of self-nucleic acids, loss of immune tolerance, immune-complex deposition, and sustained type I interferon (IFN-I)-dominated inflammation. Although NETs remain the best-established extracellular trap population in lupus, accumulating evidence suggests that METs may also amplify disease in a tissue- and context-dependent manner [124]. In lupus, the key pathogenic issue is not simply trap formation, but the imbalance between extracellular trap generation and clearance: when macrophage-derived chromatin structures persist, they may prolong exposure to extracellular DNA, oxidized mitochondrial DNA, citrullinated proteins, and other immunogenic cargo capable of fueling innate nucleic-acid sensing, complement activation, and autoantibody production [28]. Preclinical work in Fc gamma receptor IIb (FcγRIIb)-deficient lupus models supports this concept. Under inflammatory conditions, MET formation can be induced, but it is reduced by spleen tyrosine kinase (Syk) or p38 MAPK inhibition. This reduction is accompanied by improved inflammatory markers and lower disease severity [125]. At the organ level, this mechanism is particularly plausible in lupus nephritis, where renal macrophage enrichment is closely linked to prognosis and extracellular trap structures have been described in injured renal tissue, suggesting that macrophage trap programs may participate in local tissue damage and chronic fibroinflammatory remodeling [28].

#### 4.4.3. Role of METs in the Pathogenesis of Type 1 Diabetes

Type 1 diabetes mellitus is an autoimmune disease characterized by T-cell-mediated destruction of pancreatic β-cells, and increasing evidence suggests that intestinal inflammation and macrophage dysfunction participate in the early events that precede overt hyperglycemia [126]. In this context, METs appear to link gut immune dysregulation with pancreatic autoimmunity [127]. Studies in non-obese diabetic (NOD) mice show that PAD4-dependent MET formation is markedly increased in the colon. This increase is mainly associated with M1-polarized macrophages and promotes transcription of the chemokine C-X-C motif chemokine ligand 10 (CXCL10) [127]. By binding to its receptor, C-X-C motif chemokine receptor 3 (CXCR3), on T cells, CXCL10 promotes the recruitment and migration of gut-derived proinflammatory T-cell subsets toward pancreatic islets. These include T helper 1 (Th1) and cytotoxic T cell 1 (Tc1) cells. This process intensifies insulitis and accelerates β-cell injury [127,128]. The pathogenic importance of this pathway is supported by findings that PAD4 deficiency reduces intestinal M1 macrophage accumulation, suppresses colonic MET formation, attenuates inflammatory T-cell trafficking, and delays diabetes progression [127]. Similarly, pharmacologic blockade of the CXCL10/CXCR3 axis alleviates colonic inflammation, reduces peripheral and pancreatic lymph node infiltration by proinflammatory T cells, and ameliorates insulitis [127,128].

#### 4.4.4. Macrophage Extracellular Traps in ANCA-Associated Vasculitis

Antineutrophil cytoplasmic antibody (ANCA)-associated vasculitis (AAV) is a necrotizing small-vessel vasculitis defined by autoantibodies against myeloperoxidase (MPO) or proteinase 3 (PR3) [129]. Although neutrophil extracellular traps remain the best-established extracellular trap population in AAV, emerging evidence suggests that macrophages may also contribute through MET-like structures, particularly in renal lesions [28]. Monocytes and macrophages are abundant in glomerular crescents. In myeloperoxidase–antineutrophil cytoplasmic antibody (MPO-ANCA) glomerulonephritis, histological studies have identified MPO-containing CD68-positive macrophages forming extracellular trap-like structures. This suggests that at least some lesional macrophages may release endogenous MPO and extracellular chromatin, rather than simply clearing neutrophil-derived material [130]. This distinction is mechanistically important because MET-associated DNA and MPO may act locally as damage-associated molecular patterns (DAMPs) and autoantigenic material [130]. In this way, they could sustain nucleic-acid sensing, type I interferon amplification, endothelial injury, and crescentic inflammation. However, in mixed inflammatory lesions, passive uptake of neutrophil-derived material remains an important interpretive limitation. [130]. Clinically, this concept is relevant because macrophage-rich renal inflammation is linked to more severe kidney injury and poorer outcomes. Preclinical data also suggest that targeting the extracellular trap–DNA-sensing axis may be beneficial. In myeloperoxidase–antineutrophil cytoplasmic antibody-associated vasculitis (MPO-AAV) models, DNase I reduces extracellular MPO and ET burden. Inhibition of STING or JAK1/2 signaling also improves disease manifestations, including pulmonary hemorrhage [131,132,133].

#### 4.4.5. METs in Inflammatory Bowel Disease

In Crohn’s disease, which is characterized by transmural intestinal inflammation, epithelial barrier disruption, and a dominant macrophage- and T-cell-driven immune response, the role of METs is biologically plausible but still not fully defined [134,135,136]. Evidence from TNBS-induced murine colitis, a commonly used Crohn’s-like model, shows increased extracellular trap formation in ulcerated colonic tissue and in macrophage populations recovered from inflammatory compartments, indicating that macrophages participate in excessive intestinal immune activation [136]. In this setting, PAD2- and PAD4-associated pathways appear to contribute to trap formation, suggesting that METs may amplify mucosal inflammation by sustaining extracellular DNA exposure and macrophage-derived inflammatory signaling [136]. However, the precise pathogenic weight of METs in Crohn’s disease remains less certain than in ulcerative colitis, and future studies will need to distinguish direct MET-dependent effects from broader PAD-driven macrophage activation programs.

In ulcerative colitis (UC), the evidence for MET involvement is stronger and more mechanistically developed [137]. UC is a chronic mucosal inflammatory disease in which repeated cycles of epithelial injury and repair can progress toward fibrosis and persistent barrier dysfunction [138]. Recent preclinical work suggests that PAD4-driven METs are increased in colonic tissue and contribute to both intestinal inflammation and fibro-inflammatory remodeling [137,139]. In this context, osteopontin, encoded by *secreted phosphoprotein 1* (*SPP1*), is best viewed as a profibrotic mediator released by macrophages. It promotes fibroblast activation and ECM deposition, rather than acting only as a general inflammatory marker [137]. Chronic inflammation appears to increase PAD4 activity in macrophages. This supports MET formation and increases osteopontin expression through RELA/signal transducer and activator of transcription 1 (STAT1)-related transcriptional programs. As a result, it promotes myofibroblast transition and collagen accumulation [137]. In parallel, METs may also aggravate epithelial injury and barrier dysfunction, making them relevant not only to inflammation but also to structural remodeling in UC [139]. Accordingly, PAD4 inhibition has emerged as a mechanistically attractive strategy because it may reduce MET burden, barrier injury, and fibrosis simultaneously [137].

**Figure 6:**
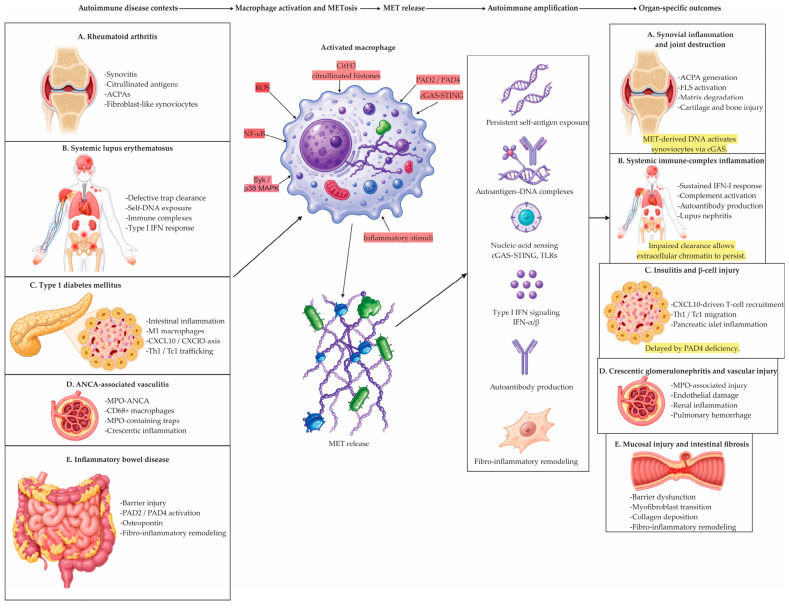
Macrophage extracellular traps as amplifiers of autoimmune inflammation. The figure summarizes how MET-derived DNA–protein complexes may contribute to autoantigen exposure, nucleic acid sensing, cytokine production, fibro-inflammatory remodeling, and organ-specific autoimmune injury. Abbreviations: METs, macrophage extracellular traps; RA, rheumatoid arthritis; SLE, systemic lupus erythematosus; T1D, type 1 diabetes; AAV, ANCA-associated vasculitis; ANCA, anti-neutrophil cytoplasmic antibody; IBD, inflammatory bowel disease; UC, ulcerative colitis; PAD, peptidylarginine deiminase; cGAS, cyclic GMP–AMP synthase; STING, stimulator of interferon genes.

The proposed role of METs as tissue-embedded amplifiers of autoimmune inflammation, including their upstream triggers, downstream immune effects, and organ-specific consequences in rheumatoid arthritis, systemic lupus erythematosus, type 1 diabetes, ANCA-associated vasculitis, and inflammatory bowel disease, is summarized in Figure 6.

### 4.5. METs in Diverse Organ-Specific Disorders

In a broad range of other non-infectious diseases, METs are increasingly emerging as pathogenic effectors that connect sterile inflammation, oxidative and metabolic stress, and tissue remodeling across multiple organs [2]. Rather than acting only as byproducts of macrophage activation, METs appear to function as active amplifiers of injury by delivering extracellular DNA, histones, proteases, and other danger-associated signals into already vulnerable microenvironments [140]. Through these mediators, METs can intensify epithelial and endothelial damage, disrupt barrier integrity, promote inflammatory cytokine release, activate DNA-sensing and NF-κB-related pathways, and drive maladaptive crosstalk between macrophages and structural cells such as fibroblasts, mesothelial cells, stellate cells, and parenchymal cells [87]. In this way, METs have been linked to disease progression in conditions as diverse as severe acute pancreatitis, Hirschsprung-associated enterocolitis, cerebral ischemia–reperfusion injury, diabetic retinopathy, progressive myopia, liver fibrosis, and peritoneal fibrosis [141,142,143]. Although the dominant downstream consequences differ by organ, ranging from immune dysregulation and neuronal injury to vascular leakage, fibrosis, and pathological remodeling, the recurring pattern is that METs translate local stress signals into sustained inflammatory and tissue-destructive responses. Collectively, these observations support the view that METs represent a common mechanistic bridge between innate immune activation and organ damage, and they further highlight MET-targeted strategies as a promising area for future therapeutic development.

#### 4.5.1. METs in Acute Pancreatitis

Severe acute pancreatitis is a highly dynamic inflammatory disorder characterized by an early systemic inflammatory response followed by a compensatory immunosuppressive phase, and this shift is closely linked to infectious complications, multi-organ injury, and poor clinical outcome [144]. Within this context, macrophages represent a dominant immune cell population and appear to act as central regulators of the transition between proinflammatory and immunosuppressive states [145]. Emerging evidence indicates that METs are important mediators of this process [141]. In experimental severe acute pancreatitis induced by pancreatic duct ligation and caerulein stimulation, MET formation is markedly increased in the injured pancreas and exceeds NET abundance, while circulating extracellular DNA is elevated in both mice and patients, supporting the clinical relevance of this pathway [141]. At the signaling level, ROS-dependent gasdermin D (GSDMD) signaling promotes MET formation during pancreatitis. These traps then influence disease progression through a dual immunoregulatory mechanism. [141]. First, METs stimulate acinar cells to release interleukin-33 (IL-33). They do this by activating the cyclic GMP-AMP synthase–stimulator of interferon genes–gasdermin D (cGAS–STING–GSDMD) pathway and promoting pyroptosis [141,146]. Second, MET-derived proteases, including MMP-12, cleave extracellular IL-33 into highly bioactive forms, which then engage the ST2 receptor and drive type 2 immune responses characterized by increased Th2 cells, regulatory T cells, and M2 macrophages, thereby contributing to compensatory anti-inflammatory response syndrome [141]. This sequence places METs at the interface between tissue injury, innate immune activation, and maladaptive immunosuppression. Importantly, therapeutic clearance of METs with DNase I or inhibition of trap formation with Cl-amidine attenuates IL-33 release, reduces Th2 activation, and alleviates disease severity in experimental models [141].

#### 4.5.2. Macrophage Extracellular Traps in Hirschsprung-Associated Enterocolitis

Hirschsprung-associated enterocolitis (HAEC) is the most frequent and most serious complication of Hirschsprung disease, characterized by recurrent intestinal inflammation, substantial morbidity, and a continuing risk of severe clinical deterioration [147]. Recent evidence suggests that METs may actively participate in this process rather than merely reflect macrophage activation [142]. In HAEC, the inflammatory milieu appears to be shaped in part by pyroptotic cell death, which releases danger signals that recruit macrophages and drive extracellular trap formation [142,147]. Among these signals, HMGB1 has emerged as a key upstream mediator: once released from the pyroptotic microenvironment, it promotes MET formation predominantly through toll-like receptor 4 (TLR4)-dependent activation of the p38 MAPK and p65 NF-κB pathways in macrophages [142]. Functionally, these METs then amplify local inflammation and worsen mucosal injury by enhancing the crosstalk between macrophages and colonic epithelial cells, thereby increasing epithelial damage and perpetuating disease progression [142].

#### 4.5.3. Extracellular Trap Formation in Post-Ischemic Brain Injury

Cerebral ischemia–reperfusion injury is a major determinant of secondary damage after recanalization therapy for acute ischemic stroke, because restoration of blood flow, although essential for tissue salvage, can also intensify oxidative stress, inflammatory signaling, and neuronal loss [148]. In this setting, the macrophage-lineage extracellular trap response in the brain appears to be mediated predominantly by microglia, which have been identified as the principal ET-producing cells in the cerebral parenchyma 24 h after reperfusion [143,149]. At the metabolic level, oxygen-glucose deprivation/reoxygenation induces microglial ET formation. This is accompanied by mitochondrial reactive oxygen species (mtROS) accumulation and a shift toward glycolytic metabolism. It also involves increased succinate dehydrogenase (SDH) activity and upregulation of the SDHA subunit, suggesting that metabolic rewiring is directly linked to trap release [143,150]. This is biologically important because succinate oxidation fuels mitochondrial oxidative stress, which in turn promotes extracellular trap formation and amplifies neuronal injury during reperfusion [143]. Consistent with this model, dimethyl malonate (DMM), a competitive succinate dehydrogenase inhibitor, suppresses microglial extracellular trap formation by limiting succinate oxidation and mtROS generation. This is associated with reduced neuronal damage and better neurological outcomes, with effects comparable to those of the ET inhibitor BB-Cl-amidine [143,151].

#### 4.5.4. METs in Ocular Diseases

Diabetic retinopathy is one of the most severe microvascular complications of diabetes and, particularly in its proliferative stage, a major cause of irreversible vision loss [152]. Increasingly, it is viewed not simply as a vascular lesion, but as a neurovascular-inflammatory disorder in which chronic hyperglycemia, oxidative stress, breakdown of the blood–retinal barrier, and innate immune activation jointly drive retinal injury [153]. In this context, METs appear to aggravate retinal microangiopathy by directly converting endothelial stress into cell death and inflammatory dysfunction [154]. Mechanistically, the process begins with endocytosis of METs by human retinal microvascular endothelial cells rather than a purely superficial receptor interaction [154]. Once internalized, METs disrupt cytoskeletal organization, fragment F-actin, and weaken the anchorage of endothelial cells to the extracellular matrix [154]. This loss of structural attachment is biologically critical because retinal endothelial survival depends on continuous integrin-mediated activation of focal adhesion kinase (FAK) and its downstream effector AKT, which normally preserve cell polarity, barrier integrity, and resistance to apoptosis [154,155]. When MET exposure reduces FAK phosphorylation and suppresses AKT signaling, endothelial cells lose this anchorage-dependent survival program and become susceptible to anoikis, a form of apoptosis triggered specifically by detachment from the matrix [154,155]. This transition is reinforced by oxidative and mitochondrial injury, as MET-treated cells accumulate reactive oxygen species, undergo mitochondrial fragmentation, release cytochrome c, and activate caspase-3, indicating that METs do not merely destabilize the endothelium mechanically, but actively initiate a mitochondrial death cascade [154]. At the same time, MET internalization can reprogram endothelial cells toward a pro-inflammatory state through the sphingolipid pathway. Upregulation of sphingosine kinase 1/2 (SPHK1/2) increases sphingosine-1-phosphate (S1P) production, while stronger sphingosine-1-phosphate receptor 2 (S1PR2) signaling promotes NF-κB activation, cytokine release, vascular leakage, and a pro-angiogenic environment [154,156]. In this way, METs link structural detachment, oxidative stress, inflammatory lipid signaling, and endothelial death into a single pathogenic sequence that culminates in blood–retinal barrier failure and retinal vascular dysfunction [154]. The observation that DNase I or blockade of endocytosis attenuates these downstream events further supports the idea that the DNA scaffold of METs acts as a functional danger signal capable of driving diabetic retinal injury independently of systemic glycemic changes [2,154].

Myopia, especially high myopia, is a major global cause of visual morbidity, and its progression is closely linked to scleral remodeling, extracellular matrix disorganization, and biomechanical weakening of the posterior eye wall [157]. Recent work indicates that macrophages participate in this remodeling process not only through conventional inflammatory signaling but also through MET release within the sclera [158]. In a lens-induced myopia model, scleral METs were markedly increased, and inhibition of MET release, either genetically or with lactoferrin, significantly attenuated myopia progression, supporting a functional rather than merely associative role [158,159]. At the upstream level, scleral hypoxia appears to be a central trigger, but hypoxia alone is not sufficient. Instead, hypoxia-activated platelets cooperate with macrophages to induce autophagy-dependent MET formation, with hypoxia-inducible factor-1α (HIF-1α) acting as a key regulator [158]. These METs then promote activation of human scleral fibroblasts and their differentiation into myofibroblasts, providing a plausible link between macrophage stress responses and the fibrotic-remodeling program that underlies progressive axial elongation [158,160]. Taken together, these findings position METs as a newly recognized bridge between hypoxic stress, platelet-driven innate immune activation, and structural scleral remodeling in myopia.

#### 4.5.5. Role of METs in Hepatic Fibrogenesis

Liver fibrosis is a chronic progressive wound-healing disorder that develops when repeated hepatic injury drives persistent inflammation, activation of hepatic stellate cells, and excessive extracellular matrix deposition, ultimately predisposing to cirrhosis and liver failure [161]. In this setting, METs are increasingly emerging as active profibrotic effectors rather than simple markers of macrophage activation [162]. Recent experimental data support a hepatocyte–macrophage–hepatic stellate cell (HSC) axis in liver fibrosis. Early during fibrogenesis, injured hepatocytes release interleukin-25 (IL-25), which binds to interleukin-17 receptor B (IL-17RB) on macrophages [162]. This triggers ROS production, lysosomal activation, and MET formation [162]. These METs then stimulate HSCs, reinforcing fibrogenic activation and accelerating liver fibrosis [162]. Mechanistically, this places METs between hepatocyte injury and stellate-cell activation [162,163]. A similar principle has also been observed in toxicologic fibrosis models, where the brominated flame retardant 1,2-bis(2,4,6-tribromophenoxy)ethane induces hepatic fibrosis through MET–stellate cell crosstalk, further supporting the broader concept that METs can translate inflammatory or toxic stress into stellate-cell activation [164].

#### 4.5.6. Macrophage Extracellular Traps in Peritoneal Fibrotic Remodeling

Peritoneal fibrosis is a major long-term complication of peritoneal dialysis in which repeated exposure to bioincompatible, especially high-glucose, dialysis fluid drives persistent sterile inflammation, neoangiogenesis, mesothelial injury, and progressive submesothelial matrix deposition, ultimately impairing ultrafiltration and peritoneal membrane function [165]. Within this setting, METs are emerging as active profibrotic mediators rather than simple markers of macrophage activation [166]. In experimental models using high-glucose peritoneal dialysis fluid, MET formation is markedly increased in the peritoneum. This is accompanied by higher expression of interleukin-1β (IL-1β), tumor necrosis factor-α (TNF-α), vascular endothelial growth factor (VEGF), and collagen I. Increased vascular density and peritoneal thickening are also observed, linking macrophage trap formation with both inflammatory and structural remodeling [166]. At the cellular level, high-glucose dialysis fluid can directly induce MET release through a partially ROS-dependent process. These METs then act on peritoneal mesothelial cells and activate the ROS/TGF-β/Smad2/3 axis, a key pathway in mesothelial-to-mesenchymal transition (MMT) and fibrogenesis [166,167]. In other words, METs appear to translate macrophage stress responses into a profibrotic signal that converts mesothelial cells from a barrier-maintaining phenotype into matrix-producing, mesenchymal-like effector cells, thereby promoting chronic peritoneal membrane remodeling.

In summary, METs emerge across non-infectious diseases as active immunopathological effectors that can convert macrophage activation into sustained tissue injury, chronic inflammation, autoimmunity, fibrosis, vascular damage, and maladaptive repair. Although MET formation is physiologically useful during host defense, excessive or insufficiently cleared METs expose tissues to extracellular DNA, citrullinated histones, oxidized mitochondrial DNA, proteases, and other danger-associated signals that activate inflammatory pathways such as cGAS–STING, NF-κB, PAD4/CitH3, ROS-dependent signaling, inflammasome-related mechanisms, and profibrotic stromal crosstalk. This pathogenic principle is evident in kidney injury, respiratory disease, metabolic inflammation, atherosclerosis, autoimmune disorders, inflammatory bowel disease, pancreatitis, neurological ischemia–reperfusion injury, ocular remodeling, hepatic fibrosis, and peritoneal fibrosis. Across these conditions, METs do not act as a uniform mechanism, but rather as tissue-specific amplifiers whose consequences depend on the local microenvironment, dominant macrophage phenotype, associated structural cells, and efficiency of extracellular trap clearance. Therefore, therapeutic strategies aimed at limiting MET formation, promoting extracellular DNA degradation, inhibiting PAD-dependent histone citrullination, modulating ROS or DNA-sensing pathways, or interrupting MET-driven stromal activation may represent an important translational direction in inflammatory and fibrotic medicine, as summarized in Table 3.

### 4.6. METs in Oncology

Cancer develops within a dynamically evolving inflammatory microenvironment in which innate immune cells, stromal elements, and tumor cells engage in continuous bidirectional crosstalk [168]. In this context, METs are increasingly recognized as functionally relevant components of the tumor microenvironment rather than incidental byproducts of macrophage activation [15]. By releasing extracellular DNA, proteases, cytokines, chemokines, and other bioactive mediators, METs can intensify chronic inflammation, disrupt immune homeostasis, and create conditions that favor tumor initiation and progression, particularly in malignancies arising on a background of persistent inflammatory injury [2,15]. Within established tumors, METs have been identified in several solid cancers, including colorectal cancer and pancreatic ductal adenocarcinoma, where higher MET burden generally correlates with more aggressive disease and poorer clinical outcome [169,170]. At the molecular level, METs may promote malignant progression through several mechanisms. They can support ECM degradation through proteases such as MMP-9 and MMP-12. They may also stimulate angiogenesis through VEGF and fibroblast growth factor (FGF), while reinforcing inflammatory signaling through HMGB1, toll-like receptor 4 (TLR-4), and NF-κB. Together, these effects may facilitate invasion, metastatic spread, and tumor-supportive tissue remodeling [15].

At the same time, the biological role of METs in oncology is clearly context-dependent, because they may also exert tumor-restraining effects under selected immune conditions [2,15]. Their functional orientation appears to be shaped largely by the immune status of the tumor microenvironment. In early or more immunogenic tumors, where M1-like macrophages, cytotoxic T cells, and NK cells are relatively preserved, METs may cooperate with phagocytosis, physically restrain tumor cells, release cytotoxic mediators, and support antitumor immune surveillance [2,15]. In contrast, during tumor progression, the tumor microenvironment (TME) often becomes more immunosuppressive. This includes expansion of regulatory T cells (Tregs) and myeloid-derived suppressor cells (MDSCs), repolarization of tumor-associated macrophages (TAMs) toward an M2-like phenotype, and sustained activation of NF-κB, Janus kinase/signal transducer and activator of transcription 3 (JAK/STAT3), and MAPK signaling [171]. Under these conditions, METs more often acquire protumorigenic functions. They can amplify immune suppression, angiogenesis, and metastatic potential. They may also participate in positive feedback loops driven by transforming growth factor-β (TGF-β), C-X-C motif chemokine ligand 8 (CXCL8), and programmed death-ligand 1 (PD-L1)-associated signaling [15,170]. This duality has important translational implications, because it suggests that METs may serve not only as biomarkers of disease behavior but also as therapeutic targets, particularly through strategies aimed at inhibiting trap formation, blocking PAD-dependent programs, or reprogramming the immunosuppressive tumor microenvironment toward a more effective antitumor state.

#### 4.6.1. METs in Colorectal Cancer

Colorectal cancer (CRC) is a highly inflammation-responsive malignancy in which tumor-associated macrophages are abundant components of the microenvironment, and current evidence suggests that METs may represent one mechanism by which these cells acquire tumor-promoting activity [169,172]. In colon cancer specimens, METs are significantly enriched within the tumor core compared with adjacent non-neoplastic tissue, and high intratumoral MET burden has been associated with distant metastasis, worse cancer-specific survival, and independent adverse prognostic value [169]. Functionally, METs appear to enhance the invasive phenotype of CRC cells, rather than simply reflecting macrophage activation. In experimental systems, phorbol 12-myristate 13-acetate (PMA)-induced METs increase the invasiveness of HCT116 and SW480 cells. In turn, conditioned medium from colon cancer cells further increases MET release, suggesting a reciprocal positive-feedback loop between malignant epithelial cells and macrophages [15,169]. This interaction is biologically plausible because extracellular trap scaffolds can retain proteases and other inflammatory mediators that facilitate matrix remodeling and tumor-cell dissemination [15]. Importantly, interruption of this crosstalk with the selective PAD2 inhibitor PAD2-IN-1 suppresses MET formation and reduces liver metastasis in vivo, supporting the concept that in colorectal cancer, METs are not merely correlates of inflammation but active contributors to metastatic progression and potentially useful prognostic and therapeutic targets [169].

#### 4.6.2. METs in Hepatocellular Carcinoma

Hepatocellular carcinoma is a highly lethal malignancy that usually develops in a chronically inflamed liver and is characterized by marked immune dysregulation, therapeutic resistance, and frequent disease progression despite systemic treatment [173]. In this malignancy, macrophages are major components of the tumor microenvironment and can actively shape treatment response [174]. In this setting, METs appear to contribute to sorafenib resistance. Sorafenib exerts part of its antitumor effect by inducing ferroptosis, a form of regulated cancer cell death driven by iron-dependent lipid peroxidation, meaning that tumor cells die because oxidative damage accumulates in their membranes beyond a survivable threshold [175]. Recent experimental work suggests that, paradoxically, sorafenib can also trigger a compensatory tumor–macrophage response in which HCC cells increase IL-4 release and thereby skew surrounding macrophages toward an M2-like, tumor-supportive phenotype with enhanced PAD4-dependent MET formation [175]. These METs then blunt the antitumor effect of sorafenib by making ferroptosis harder to execute [175]. A key part of this protection involves increased expression of glutathione peroxidase 4 (GPX4), an important intracellular enzyme that neutralizes lipid peroxides. Since ferroptosis depends on the toxic accumulation of these peroxides, higher GPX4 activity can protect tumor cells from ferroptotic death [175,176]. In other words, the macrophage trap response converts drug-induced oxidative stress into an adaptive survival program for the tumor. This mechanism is clinically relevant because degrading the MET DNA scaffold with DNase I or interrupting the IL-4 signal restores sorafenib sensitivity and reduces tumor growth in preclinical models, supporting the idea that METs are not just markers of inflammation in HCC but active mediators of therapy resistance [175].

#### 4.6.3. Role of METs in Glioblastoma Progression

Glioblastoma is the most aggressive primary brain tumor in adults and is characterized by a profoundly immunosuppressive microenvironment, extensive necrosis, diffuse invasion, and poor survival [177]. Within this landscape, macrophages and microglia constitute a major immune compartment, and available evidence suggests that METs may represent a specialized macrophage response within necrotic and highly inflamed tumor regions [178,179]. A small pathological study of 15 glioblastoma specimens found that M1-type macrophages were more common in normal brain and tumor-border areas, whereas M2 macrophages predominated in the tumor core, and MET-like structures were detected mainly at the interface between viable tumor and necrotic tissue, where they co-localized with markers consistent with fibrin-rich inflammatory exudation [178]. This localization suggests that METs may participate in remodeling of the tumor–necrosis niche, potentially influencing local inflammation, matrix organization, and tumor cell behavior [178]. At the same time, an intriguing but still preliminary observation from that cohort was that the few patients with detectable METs had longer survival than those without METs, raising the possibility that, in contrast to many other cancers, METs in glioblastoma might under some conditions restrain invasion or reflect a more effective local immune response [178]. However, this interpretation remains highly tentative because the evidence is based on a very limited patient series and the underlying mechanisms have not yet been defined.

#### 4.6.4. Role of METs in PDAC Progression and Metastasis

Pancreatic ductal adenocarcinoma (PDAC) is one of the most aggressive solid malignancies, characterized by an intensely immunosuppressive microenvironment, early dissemination, and a marked tendency to metastasize to the liver [180]. In this setting, METs appear to function as active promoters of tumor progression rather than passive markers of inflammation. A particularly important upstream event is necroptosis, a regulated form of inflammatory cell death mediated by mixed lineage kinase domain-like protein (MLKL) [170]. In PDAC, increased MLKL activity has been linked to early liver metastasis, suggesting that necroptotic tumor-cell stress is not merely a by-product of aggressive disease, but part of the metastatic program itself [170]. At the signaling level, MLKL-driven necroptosis changes the interaction between tumor cells and macrophages in several ways. It increases cluster of differentiation 47 (CD47) expression on tumor cells, which weakens macrophage phagocytic clearance. At the same time, it stimulates macrophages to release METs through a C-X-C motif chemokine ligand 8 (CXCL8)-centered signaling loop [170,181]. These METs then intensify malignant behavior by amplifying CXCL8 signaling and promoting epithelial–mesenchymal transition (EMT). They also increase intercellular adhesion molecule-1 (ICAM-1)-dependent endothelial adhesion and support ECM remodeling. Together, these effects favor hematogenous spread and liver colonization [170,182]. Thus, in PDAC, MLKL-driven necroptosis should be understood as the inflammatory trigger that converts macrophages from potential antitumor effectors into MET-producing partners of metastasis, making the necroptosis–CXCL8–MET axis a mechanistically coherent and potentially targetable pathway in pancreatic cancer.

#### 4.6.5. METs in Nonfunctional Pancreatic Neuroendocrine Tumors

Nonfunctional pancreatic neuroendocrine tumors are clinically heterogeneous neoplasms for which postoperative risk stratification still relies mainly on WHO grade and TNM stage, although these parameters do not fully capture the biologic variability that determines recurrence [183]. In this setting, METs may serve as biomarkers of a more aggressive immune microenvironment. In a cohort of 135 patients with resectable nonfunctional pancreatic neuroendocrine tumors (pNETs), high macrophage infiltration and MET positivity in postoperative specimens were associated with significantly shorter recurrence-free survival (RFS). Both factors also remained independent predictors of recurrence in multivariable analysis. [184]. This is clinically important because it suggests that macrophages in these tumors are not merely passive infiltrates, but may contribute to progression through extracellular trap–associated inflammatory signaling that supports tumor growth, invasion, and recurrence [184,185]. On this basis, the authors proposed that innate immune parameters, including MET status, could complement standard clinicopathological models. This may improve prognostic stratification and help refine postoperative surveillance and treatment selection in nonfunctional pancreatic neuroendocrine tumors (NF-pNETs) [184].

**Figure 7:**
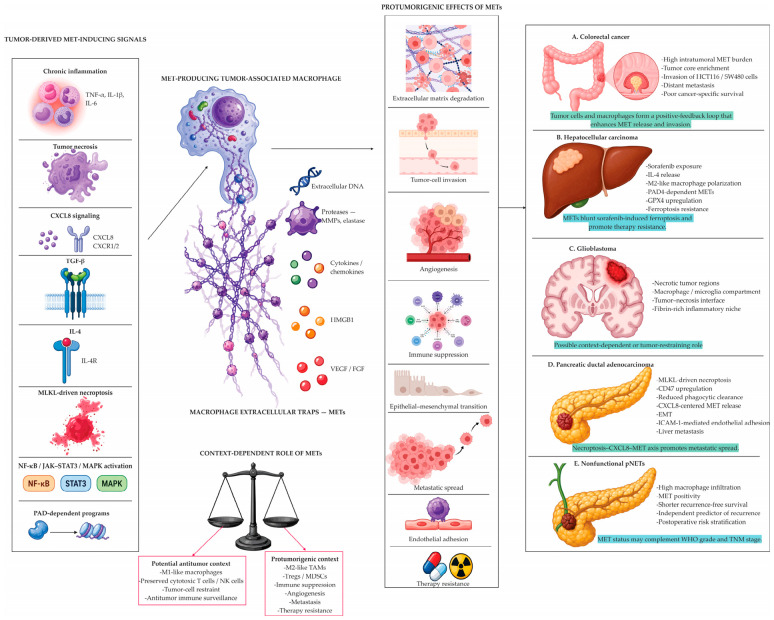
Context-dependent roles and protumorigenic effects of macrophage extracellular traps in cancer. The figure summarizes tumor-associated signals that may induce MET release and the downstream effects of METs on invasion, angiogenesis, immune suppression, metastatic spread, endothelial adhesion, and therapy resistance. Abbreviations: METs, macrophage extracellular traps; TAMs, tumor-associated macrophages; CXCL8, C-X-C motif chemokine ligand 8; TGF-β, transforming growth factor-β; IL-4, interleukin-4; MLKL, mixed lineage kinase domain-like pseudokinase; NF-κB, nuclear factor kappa B; JAK/STAT3, Janus kinase/signal transducer and activator of transcription 3; MAPK, mitogen-activated protein kinase; PAD, peptidylarginine deiminase; HMGB1, high-mobility group box 1; VEGF, vascular endothelial growth factor; FGF, fibroblast growth factor; EMT, epithelial–mesenchymal transition; ECM, extracellular matrix; PDAC, pancreatic ductal adenocarcinoma; pNET, pancreatic neuroendocrine tumor.

Figure 7 summarizes tumor-derived MET-inducing signals and the main cancer-specific consequences of MET formation.

In summary, METs are functionally relevant components of the tumor microenvironment, with effects that depend on macrophage phenotype and the broader immune context. In established immunosuppressive tumors, they more often support invasion, metastasis, immune escape, and treatment resistance. This tumor-supportive role is particularly evident in colorectal cancer, pancreatic ductal adenocarcinoma, hepatocellular carcinoma, and nonfunctional pancreatic neuroendocrine tumors, where MET burden has been linked to metastasis, recurrence, poor outcome, or reduced treatment sensitivity. However, the glioblastoma data remind us that MET biology in cancer is not uniform, and that in selected immune contexts METs may reflect a more active local inflammatory response rather than a purely harmful process. Overall, METs should be viewed as context-dependent immune platforms within cancer, with potential value as prognostic biomarkers and as therapeutic targets through strategies aimed at inhibiting PAD-dependent trap formation, degrading extracellular DNA scaffolds, blocking tumor–macrophage feedback loops, or reprogramming the immunosuppressive tumor microenvironment, as summarized in Table 4.

## 5. Conclusions

Taken together, the available evidence supports the view that METs are not merely macrophage-derived analogues of neutrophil extracellular traps, but distinct and highly context-dependent effector structures that occupy a central position at the intersection of innate immunity, tissue remodeling, and disease pathogenesis. Across infectious settings, METs may reinforce host defense by trapping pathogens and restricting their dissemination, whereas in sterile inflammatory conditions their persistence or dysregulated formation can amplify tissue injury, fibrosis, metabolic dysfunction, autoimmunity, vascular pathology, and tumor progression. A recurring theme across disease models is that METs translate local stress signals into extracellular DNA- and protease-rich inflammatory microenvironments, thereby linking macrophage activation to chronic damage through pathways involving PAD-dependent chromatin remodeling, ROS and calcium signaling, mitochondrial dysfunction, cGAS–STING activation, and maladaptive stromal crosstalk. At the same time, major conceptual and technical challenges remain unresolved, including the rigorous lineage attribution of METs in mixed lesions, the standardization of in vivo detection, the distinction between lytic and non-lytic METosis, and the need to define when METs are true disease drivers rather than secondary correlates of severe inflammation. These gaps are particularly relevant for translation, because therapeutic strategies such as PAD inhibition, extracellular DNA degradation, blockade of DNA-sensing pathways, or modulation of upstream macrophage programs will only succeed if applied with sufficient biological precision. Future research should therefore move beyond descriptive observations toward lineage-resolved mechanistic studies, standardized biomarkers, and disease-specific intervention models that clarify when, where, and how METs should be targeted. Such advances will be essential for determining whether METs can be exploited not only as biomarkers of immune dysregulation, but also as actionable therapeutic nodes across a wide spectrum of inflammatory, fibrotic, autoimmune, metabolic, and malignant diseases.

## Figures and Tables

**Table 1:** Integrated biological and clinical relevance of macrophage extracellular traps.

Aspect	Main Biological Insight	Mechanistic Interpretation	Clinical Significance/Therapeutic Implication	References
Macrophage defense function	METs expand macrophage activity beyond phagocytosis and cytokine release.	Activated macrophages externalize DNA-based structures that immobilize microorganisms and concentrate antimicrobial proteins.	METs may support host defense when pathogen clearance by phagocytosis alone is insufficient.	[2,3,12,13]
Molecular composition	METs consist mainly of extracellular DNA, histones, antimicrobial enzymes, and macrophage-associated proteins.	PAD2/PAD4-mediated histone citrullination facilitates chromatin relaxation and scaffold formation.	Components such as citrullinated histones, extracellular DNA, CD68, MPO, and MMPs may help identify METs in tissue and experimental samples.	[4,15,16,17,18,22,23,24]
Regulation of METosis	MET formation is induced by infectious and sterile inflammatory stimuli.	ROS generation, calcium signaling, MAPK pathways, autophagy-related signaling, inflammasome activation, and mitochondrial stress may contribute depending on context.	Targeting upstream regulators such as NADPH oxidase, PAD enzymes, calcium-dependent pathways, or inflammasome signaling may reduce harmful MET formation.	[25,26,27,28,29,30,31,32,36,37,38,39,40,41,42,43,44]
Lytic and vital-like METosis	MET release may occur with terminal cell injury or with temporary preservation of macrophage viability.	Lytic METosis involves membrane permeabilization and chromatin discharge, whereas vital-like forms may involve regulated DNA export and membrane repair.	Distinguishing harmful lytic METosis from potentially controlled DNA release may be important for therapeutic modulation.	[15,18,32,42,43]
Polarization and disease amplification	METs are more closely associated with M1-polarized inflammatory macrophages than with M2-like reparative macrophages.	Pro-inflammatory cytokines, oxidative burst, and calcium influx favor MET formation, while METs may further reinforce M1 polarization.	Persistent METosis may sustain chronic inflammation, tissue damage, fibrosis, vascular injury, autoimmunity, and tumor-promoting inflammation.	[10,12,16,29,36,45,46,47,48]

**Table 2:** Protective and pathogenic roles of macrophage extracellular traps in infectious diseases.

Infectious Context	Dominant MET Function	Main Mechanistic Pattern	Clinical Significance/Therapeutic Implication	References
Gram-positive bacteria	Extracellular bacterial immobilization, local killing, and containment of tissue spread.	Often ROS-dependent, involving NADPH oxidase activity, antimicrobial proteins, elastase, MPO, histones, MAPK signaling, calcium entry, and PAD4 in selected models.	MET enhancement may support bacterial clearance in difficult infections such as *S. aureus* and *C. perfringens*, but tissue context remains crucial.	[3,22,39,49,51,53,55]
Maternal–fetal and mucosal infection	Local containment of pathogens such as group *B. Streptococcus* and uropathogenic *E. coli*.	Oxidative burst, extracellular DNA release, MMP-rich trap formation, and in some settings inflammasome-associated pathways.	Protective containment may coexist with tissue vulnerability, including fetal membrane weakening or reproductive dysfunction.	[22,28,46,52,53,58,59]
Gram-negative airway and periodontal pathogens	Restriction of bacterial persistence and dissemination in chronically exposed tissues.	ROS-dependent METosis in nontypeable *H. influenzae*; calcium-driven, ROS-independent METosis in *P. gingivalis*; calcium–PAD2 signaling in *P. aeruginosa*.	Repeated MET formation may contribute to COPD-related matrix injury, periodontal inflammation, and chronic tissue damage.	[33,64,65,69,70]
Mycobacteria and atypical bacteria	Mainly physical containment, with variable effects on microbial killing.	ESX-1/ESAT-6-associated injury in *M. tuberculosis*; calcium-dependent trap formation in rapid-growing nontuberculous mycobacteria; ROS-dependent killing in *Mycoplasma bovis*.	Therapeutic modulation must distinguish beneficial containment from pathogen-driven cell injury and persistence.	[20,27,40,71,72,73,74]
Fungi and parasites	Capture of larger or invasive organisms that are poorly suited to rapid phagocytic clearance.	Non-canonical METosis in *Candida albicans*; autophagy/ROS-related responses to aflatoxin B1; parasite-specific MAPK or immune-evasion pathways.	METs may limit fungal and parasitic spread, but pathogens can degrade or suppress traps to escape immune control.	[13,19,35,41,48,76,77]

**Table 3:** Pathogenic relevance and therapeutic implications of macrophage extracellular traps in non-infectious diseases.

Disease Setting	Dominant MET-Related Mechanism	Main Pathological Consequence	Clinical Significance/Therapeutic Implication	References
Kidney injury and crystal-associated renal disease	PAD4/CitH3-dependent METosis, heme–platelet–MAC1 signaling, ROS production, and crystal-induced macrophage activation.	Tubular injury, sterile inflammation, renal dysfunction, and amplification of crystal-associated tissue damage.	MET inhibition with agents such as zafirlukast, lactoferrin, or strategies targeting PAD4, ROS, and extracellular DNA may reduce acute kidney injury and inflammatory renal damage.	[84,85,86,87,88,89,90,91,93,94,95]
Respiratory diseases	Smoke-, infection-, IL-33/ST2-, PAD-, ROS-, and neutrophil elastase-driven MET formation in airway macrophages.	Persistent airway inflammation, protease-rich tissue injury, extracellular matrix degradation, mucus obstruction, and remodeling.	DNase 1, PAD inhibition, IL-33/ST2 blockade, and antiprotease strategies may help reduce extracellular trap burden and proteolytic lung damage.	[96,97,98,99,100,101,102]
Metabolic and vascular disorders	TNF-α-, PAD2/PAD4-, PI3K/AKT-, LPC-, and lipid stress-related METosis in adipose tissue and atherosclerotic plaques.	Adipose inflammation, insulin resistance, plaque inflammation, thrombus organization, and atherothrombotic progression.	Targeting METosis may improve metabolic inflammation and may be particularly relevant in advanced atheroinflammatory disease.	[103,104,105,106,107,108,109,110,111]
Autoimmune and inflammatory bowel diseases	Persistent extracellular exposure of citrullinated chromatin, oxidized DNA, autoantigenic cargo, and activation of cGAS–STING, PAD, Syk/p38 MAPK, CXCL10/CXCR3, and profibrotic pathways.	Loss of immune tolerance, autoantibody formation, synovial destruction, lupus-related inflammation, insulitis, vasculitic injury, intestinal barrier dysfunction, and fibrosis.	PAD inhibition, cGAS–STING blockade, DNase-based strategies, and modulation of CXCL10/CXCR3 or JAK/STING signaling may be therapeutically relevant in selected diseases.	[17,25,79,112,113,114,115,116,117,118,119,120,121,122,123,124,125,126,127,128,129,130,131]
Organ-specific sterile inflammatory injury	MET-driven cGAS–STING–GSDMD activation, MMP-12-mediated IL-33 processing, HMGB1/TLR4/p38/NF-κB signaling, and succinate–mtROS metabolic rewiring.	Severe pancreatitis progression, compensatory immunosuppression, intestinal epithelial injury, neuronal damage after reperfusion, and persistent tissue inflammation.	DNase I, Cl-amidine, metabolic inhibition of succinate oxidation, or blockade of upstream inflammatory signals may attenuate organ injury.	[133,134,135,136,137,138,139,140,141,142,143]
Fibrotic, ocular, and remodeling disorders	MET internalization, endothelial anoikis, sphingolipid inflammatory signaling, platelet–hypoxia–autophagy pathways, IL-25/IL-17RB signaling, and ROS/TGF-β/Smad2/3 activation.	Retinal vascular dysfunction, progressive myopia, hepatic stellate cell activation, mesothelial-to-mesenchymal transition, collagen deposition, and chronic fibrosis.	Blocking MET release, trap uptake, ROS signaling, or MET-driven stromal activation may limit pathological remodeling and fibrosis.	[144,145,146,147,148,149,150,151,152,153,154,155,156,157,158,159]

**Table 4:** Context-dependent roles and therapeutic implications of macrophage extracellular traps in cancer.

Cancer Setting	Dominant MET-Related Mechanism	Main Biological Consequence	Clinical Significance/Therapeutic Implication	References
General tumor microenvironment	METs release extracellular DNA, proteases, cytokines, chemokines, VEGF, FGF, HMGB1, and inflammatory mediators.	Chronic inflammation, ECM degradation, angiogenesis, immune imbalance, invasion, and metastatic remodeling.	METs may serve as biomarkers of aggressive inflammation-driven tumor behavior and as targets for trap inhibition or immune reprogramming.	[2,12,160,161,162,163]
Colorectal cancer	Reciprocal crosstalk between CRC cells and macrophages increases MET release; PAD2-dependent programs appear important.	Increased tumor-cell invasiveness, liver metastasis, and worse cancer-specific survival.	PAD2 inhibition may reduce MET formation and metastatic spread, supporting METs as prognostic and therapeutic targets.	[12,161,164]
Hepatocellular carcinoma	Sorafenib-induced IL-4 signaling promotes M2-like macrophage polarization and PAD4-dependent MET formation.	METs protect tumor cells from ferroptosis through GPX4-associated resistance mechanisms.	DNase I or IL-4 pathway interruption may restore sorafenib sensitivity in preclinical models.	[165,166,167,168]
Glioblastoma	MET-like structures appear mainly at the viable tumor–necrosis interface, associated with inflammatory and fibrin-rich regions.	Possible modulation of necrotic niche inflammation and local tumor behavior; prognostic meaning remains uncertain.	Current evidence is preliminary and requires validation in larger cohorts with mechanistic analysis.	[169,170,171]
Pancreatic ductal adenocarcinoma	MLKL-driven necroptosis increases CD47 expression and stimulates CXCL8-centered macrophage MET release.	Reduced macrophage clearance, EMT, endothelial adhesion, ECM remodeling, hematogenous spread, and liver metastasis.	Targeting the necroptosis–CXCL8–MET axis may offer a rational strategy to limit metastatic progression.	[162,172,173,174]
Nonfunctional pancreatic neuroendocrine tumors	High macrophage infiltration and MET positivity identify a more aggressive immune microenvironment.	Shorter recurrence-free survival and independent prediction of postoperative recurrence.	MET assessment may complement WHO grade and TNM stage for postoperative risk stratification and surveillance planning.	[175,176,177]

## Data Availability

As this is a review article, no new data were created or analyzed. All data supporting the findings of this study are available in the cited literature. Therefore, a data availability statement is not applicable.

## Abbreviations

The following abbreviations are used in this manuscript:

AAT	α1-antitrypsin
AAV	ANCA-associated vasculitis
ACPAs	Anti-citrullinated protein antibodies
AKI	Acute kidney injury
AKT	Protein kinase B
AMPK	AMP-activated protein kinase
ANCA	Antineutrophil cytoplasmic antibody
*B. licheniformis*	*Bacillus licheniformis*
*B. subtilis*	*Bacillus subtilis*
BAK	BCL-2 antagonist/killer 1
BAX	BCL-2-associated X protein
BB-Cl-amidine	N-α-benzoyl-N5-(2-chloro-1-iminoethyl)-L-ornithine amide
BCL-2	B-cell lymphoma 2
*C. albicans*	*Candida albicans*
*C. perfringens*	*Clostridium perfringens*
cAMP	Cyclic adenosine monophosphate
CD18	Cluster of differentiation 18
CD47	Cluster of differentiation 47
CD68	Cluster of differentiation 68
cGAS	Cyclic GMP-AMP synthase
CIRP	Cold-inducible RNA-binding protein
CitH3	Citrullinated histone H3
COPD	Chronic obstructive pulmonary disease
CpG	Cytosine-phosphate-guanine
CRAMP	Cathelicidin-related antimicrobial peptide
CRC	Colorectal cancer
CREB1	cAMP response element-binding protein 1
CRTC2	CREB-regulated transcription coactivator 2
CXCL8	C-X-C motif chemokine ligand 8
CXCL10	C-X-C motif chemokine ligand 10
CXCR3	C-X-C motif chemokine receptor 3
DAMPs	Damage-associated molecular patterns
DMM	Dimethyl malonate
DNA	Deoxyribonucleic acid
DNase	Deoxyribonuclease
DNase I/DNase 1	Deoxyribonuclease I
DPI	Diphenyleneiodonium
dsDNA	Double-stranded deoxyribonucleic acid
*E. coli*	*Escherichia coli*
ECM	Extracellular matrix
EDTA	Ethylenediaminetetraacetic acid
ERK	Extracellular signal-regulated kinase
ERK1/2	Extracellular signal-regulated kinase 1/2
ESAT-6	6-kDa early secretory antigenic target
ESCRT	Endosomal sorting complexes required for transport
ESX-1	Early secretory antigenic target 6-kDa secretion system 1
ET	Extracellular trap
F-actin	Filamentous actin
FAK	Focal adhesion kinase
FcγRIIb	Fc gamma receptor IIb
FEV1%	Predicted forced expiratory volume in 1 second percentage
FGF	Fibroblast growth factor
GBS	Group B Streptococcus
GMP-AMP	Guanosine monophosphate–adenosine monophosphate
GPX4	Glutathione peroxidase 4
GSDMD	Gasdermin D
GSK-3β	Glycogen synthase kinase-3 beta
*H. influenzae*	*Haemophilus influenzae*
H3	Histone H3
H4	Histone H4
HAEC	Hirschsprung-associated enterocolitis
HCC	Hepatocellular carcinoma
HCT116	Human colorectal carcinoma cell line HCT116
HIF-1α	Hypoxia-inducible factor-1 alpha
HMG-CoA	3-hydroxy-3-methylglutaryl coenzyme A
HMGB1	High mobility group box 1
HOCl	Hypochlorous acid
HSC	Hepatic stellate cell
ICAM-1	Intercellular adhesion molecule-1
IFN-I	Type I interferon
IFN-γ	Interferon-gamma
IL-1	Interleukin-1
IL-1β	Interleukin-1 beta
IL-4	Interleukin-4
IL-6	Interleukin-6
IL-8	Interleukin-8
IL-10	Interleukin-10
IL-12	Interleukin-12
IL-17RB	Interleukin-17 receptor B
IL-23	Interleukin-23
IL-25	Interleukin-25
IL-33	Interleukin-33
ILC1	Group 1 innate lymphoid cells
ILC3	Group 3 innate lymphoid cells
ILCs	Innate lymphoid cells
IRS-1	Insulin receptor substrate-1
J774A.1	Murine macrophage-like cell line J774A.1
JAK	Janus kinase
JAK1/2	Janus kinase 1/2
JAK/STAT3	Janus kinase/signal transducer and activator of transcription 3 pathway
kDa	Kilodalton
KIRI	Kidney ischemia/reperfusion injury
LL-37	Cathelicidin LL-37
LPC	Lysophosphatidylcholine
LPS	Lipopolysaccharide
*M. abscessus* subsp. *massiliense*	*Mycobacterium abscessus* subspecies *massiliense*
*M. bovis*	*Mycoplasma bovis*
*M. massiliense*	*Mycobacterium massiliense*
*M. tuberculosis*	*Mycobacterium tuberculosis*
M1	Classically activated pro-inflammatory macrophage phenotype
M2	Alternatively activated anti-inflammatory/tissue-repair or tumor-supportive macrophage phenotype
MAC1	Macrophage antigen-1
MAP	Magnesium ammonium phosphate
MAPK	Mitogen-activated protein kinase
MCP-1	Monocyte chemoattractant protein-1
MDSCs	Myeloid-derived suppressor cells
MEK	Mitogen-activated protein kinase kinase
MET/METs	Macrophage extracellular trap(s)
METosis	Macrophage extracellular trap formation
microRNA	Micro-ribonucleic acid
miR-93-3p	MicroRNA-93-3p
MLKL	Mixed lineage kinase domain-like protein
MMP	Matrix metalloproteinase
MMP-9	Matrix metalloproteinase-9
MMP-12	Matrix metalloproteinase-12
MMPs	Matrix metalloproteinases
MMT	Mesothelial-to-mesenchymal transition
MPO	Myeloperoxidase
MPO-ANCA	Myeloperoxidase–antineutrophil cytoplasmic antibody
mPTP	Mitochondrial permeability transition pore
mtDNA	Mitochondrial deoxyribonucleic acid
mTOR	Mechanistic target of rapamycin
mtROS	Mitochondrial reactive oxygen species
*N. caninum*	*Neospora caninum*
NAD(P)H	Reduced nicotinamide adenine dinucleotide phosphate/reduced nicotinamide adenine dinucleotide
NADPH	Nicotinamide adenine dinucleotide phosphate
NE	Neutrophil elastase
NET/NETs	Neutrophil extracellular trap(s)
NF-pNETs	Nonfunctional pancreatic neuroendocrine tumors
NF-κB	Nuclear factor kappa B
NF-κB2	Nuclear factor kappa B2
NIK	NF-κB-inducing kinase
NK cells	Natural killer cells
NOD	Non-obese diabetic
NOX	NADPH oxidase
NQO1	NAD(P)H quinone oxidoreductase 1
NTHi	Nontypeable Haemophilus influenzae
*P. aeruginosa*	*Pseudomonas aeruginosa*
*P. gingivalis*	*Porphyromonas gingivalis*
P2X7R	Purinergic P2X7 receptor
p38	p38 mitogen-activated protein kinase
p38 MAPK	p38 mitogen-activated protein kinase
p65 NF-κB	p65 subunit of nuclear factor kappa B
PAD	Peptidylarginine deiminase
PAD2	Peptidylarginine deiminase 2
PAD2-IN-1	Peptidylarginine deiminase 2 inhibitor 1
PAD4	Peptidylarginine deiminase 4
PD-L1	Programmed death-ligand 1
PDAC	Pancreatic ductal adenocarcinoma
PI3K	Phosphoinositide 3-kinase
PMA	Phorbol 12-myristate 13-acetate
pNETs	Pancreatic neuroendocrine tumors
PPAR-γ	Peroxisome proliferator-activated receptor gamma
PR3	Proteinase 3
RAF	Rapidly accelerated fibrosarcoma
RAW264.7	Murine macrophage-like cell line RAW264.7
RELA	RELA proto-oncogene, NF-κB subunit
RFS	Recurrence-free survival
ROS	Reactive oxygen species
*S. aureus*	*Staphylococcus aureus*
*S. japonicum*	*Schistosoma japonicum*
*S. stercoralis*	*Strongyloides stercoralis*
*S. Typhimurium*	*Salmonella enterica serovar Typhimurium*
S1P	Sphingosine-1-phosphate
S1PR2	Sphingosine-1-phosphate receptor 2
SDH	Succinate dehydrogenase
SDHA	Succinate dehydrogenase complex flavoprotein subunit A
Sema4D	Semaphorin 4D
Sja-miR-71a	Schistosoma japonicum microRNA-71a
SLE	Systemic lupus erythematosus
Smad2/3	SMAD family member 2/3
SPHK1/2	Sphingosine kinase 1/2
SPP1	Secreted phosphoprotein 1
sST2	Soluble suppression of tumorigenicity 2
ST2	Suppression of tumorigenicity 2 receptor
STAT1	Signal transducer and activator of transcription 1
STAT3	Signal transducer and activator of transcription 3
STING	Stimulator of interferon genes
SW480	Human colorectal adenocarcinoma cell line SW480
Syk	Spleen tyrosine kinase
TAMs	Tumor-associated macrophages
Tc1	Cytotoxic T cell 1 cells
TGF-β	Transforming growth factor-beta
Th1	T helper 1 cells
Th2	T helper 2 cells
THP-1	Human monocytic leukemia cell line THP-1
TLR4	Toll-like receptor 4
TME	Tumor microenvironment
TNBS	2,4,6-trinitrobenzene sulfonic acid
TNF-α	Tumor necrosis factor-alpha
TNM	Tumor–node–metastasis classification
Tregs	Regulatory T cells
UC	Ulcerative colitis
ULK1	Unc-51-like kinase 1
UPEC	Uropathogenic *Escherichia coli*
VDAC	Voltage-dependent anion channel
VEGF	Vascular endothelial growth factor
WHO	World Health Organization
